# Senna makki and other active phytochemicals: Myths and realities behind covid19 therapeutic interventions

**DOI:** 10.1371/journal.pone.0268454

**Published:** 2022-06-14

**Authors:** Naila Zaman, Nousheen Parvaiz, Rabia Farid, Afifa Navid, Ghulam Abbas, Syed Sikander Azam

**Affiliations:** Computational Biology Lab, National Center for Bioinformatics, Quaid-i-Azam University, Islamabad, Pakistan; Alagappa University, INDIA

## Abstract

This study aims to investigate the binding potential of chemical compounds of *Senna* in comparison with the experimentally tested active phytochemicals against SARS-CoV-2 protein targets to assist in prevention of infection by exploring multiple treatment options. The entire set of phytochemicals from both the groups were subjected to advanced computational analysis that explored functional molecular descriptors from a set of known medicinal-based active therapeutics followed by MD simulations on multiple SARS-CoV-2 target proteins. Our findings manifest the importance of hydrophobic substituents in chemical structures of potential inhibitors through cross-validation with the FDA-approved anti-3CL^pro^ drugs. Noteworthy improvement in end-point binding free energies and pharmacokinetic profiles of the proposed compounds was perceived in comparison to the control drug, vizimpro. Moreover, the identification of common drug targets namely; AKT1, PTGS1, TNF, and DPP4 between proposed active phytochemicals and Covid19 using network pharmacological analysis further substantiate the importance of medicinal scaffolds. The structural dynamics and binding affinities of phytochemical compounds xanthoangelol_E, hesperetin, and beta-sitosterol reported as highly potential against 3CL^pro^ in cell-based and cell-free assays are consistent with the computational analysis. Whereas, the secondary metabolites such as sennosides A, B, C, D present in higher amount in *Senna* exhibited weak binding affinity and instability against the spike protein, helicase nsp13, RdRp nsp12, and 3CL^pro^. In conclusion, the results contravene fallacious efficacy claims of *Senna* tea interventions circulating on electronic/social media as Covid19 cure; thus emphasizing the importance of well-examined standardized data of the natural products in hand; thereby preventing unnecessary deaths under pandemic hit situations worldwide.

## Introduction

Due to continuously evolving genetic makeup of the SARS-CoV-2 virus and its ability to spread rapidly, it has taken a huge toll on individuals, communities and societies across the world by ruthlessly affecting over 340 million people globally leading to 5.5 million deaths as of 20^th^, January 2022 [1]. However, several vaccines worldwide namely; Oxford–AstraZeneca, Pfizer-BioNTech, Sinopharm-BBIBP, Moderna, Sinovac, Johnson & Johnson, and mRNA-1273 (developed by Moderna Inc.), have been given authorization on emergency basis due to overwhelmed health systems that have caused widespread social and economic disruption [2]. Despite the rollout of vaccines to general public, trend in daily number of cases reported is still on the higher side signifying the end of the SARS-CoV-2 pandemic as implausible [3, 4]. One of the foremost reasons is the unavailability of vaccines especially in low-income and middle-income countries leading to a standstill to achieve global control of SARS-CoV-2 [5]. Another big reason is that a large number of people are reluctant to get a vaccine shot rather they believe in using herbal medicines as an alternative cure to SARS-CoV-2 [6]. Nevertheless, traditional medicinal plants have a vast history in treating infectious diseases. For example, malaria was treated for a very long time with *Artemisia Annua* (sweet wormwood) in China and *Cinchona Officinalis* (Cinchona tree) in South America [7]. Another example is the use of Chinese traditional medicine *Lianhuaqingwen* in the treatment of SARS-CoV-2 exhibiting inhibition of virus replication in a dose-dependent manner with IC_50_ of 411.2 ug/ml [8]. Thus, the role of herbal medicines in these unprecedented times of ongoing pandemic has resulted in a global catastrophe that cannot be ignored.

While the effectiveness of some medicinal plants has been scientifically proven, there is a global tendency for self-medication with different herbal medicines without proper scientific evidence. There has been a myth regarding the use of famous *Senna* tea in treatment of Covid19 that has eventually lead to its excessive use followed by a drastic hike in prices [9, 10]. It is generally used as an herbal tea made from *Senna* pods or leaves cultivated in different countries having different species commonly known as *Alexandrian Senna*, *Tinnevelly Senna*, *Indian Senna*, and *Sanna Makki*. The plant extracts of different species consist of many active anthraquinones and flavonoids including sennosides, aloe-emodin, rhein, iso-rhamnetin and kaempferol [11, 12]. Limitation of long term use of *Senna* is reported to be concomitant with dehydration and diarrhea [13]. Apprehensions on the propagation of misleading information about its use in Covid19 treatment were enhanced through social and mainstream media without sufficient scientific evidence [14]. It is of utmost importance to remove ambiguity about using *Senna* tea in Covid19 treatment, which can rather aggravate the symptoms by causing irritated bowl linings, dehydration, and electrolyte imbalance that can ultimately be fatal [15–17].

The scope of this study, therefore, serves to analyze potential binding of particularly those phytochemicals that have been suggested to inhibit the major protease of SARS-CoV-2, 3-Chymotrypsin-Like Protease (3CL^pro^) *in vitro* with IC_50_ 0–10 μM in comparison with the phytochemicals present in *Senna*. Inhibition of 3CL^pro^ is crucial in viral lifecycle and design of SARS-CoV and SARS-CoV-2 inhibitors [18]. It is highly conserved among the SARS-CoV viruses and displays 96% similarity with the zoonotic genome, especially the bat coronavirus [19, 20]. To further explore the possibility of chemical compounds of *Senna* to be active against other Covid19 protein targets, we elucidated its structural properties with additional essential proteins of SARS-CoV-2 namely; spike protein, helicase nsp13, and RdRp nsp12. The findings of this study will assist in distinguishing potential phytochemicals from a group of known and social media acclaimed plant based Covid19 treatments based on comparative structural dynamics. Moreover, compounds proposed in this study will hold a rationale to inhibit both PL^pro^ and 3CL^pro^ and provide avenues to use the scaffolds of these molecules in the design of more specific SARS-CoV-2 inhibitors in future.

## Methodology

**Fig 1:**
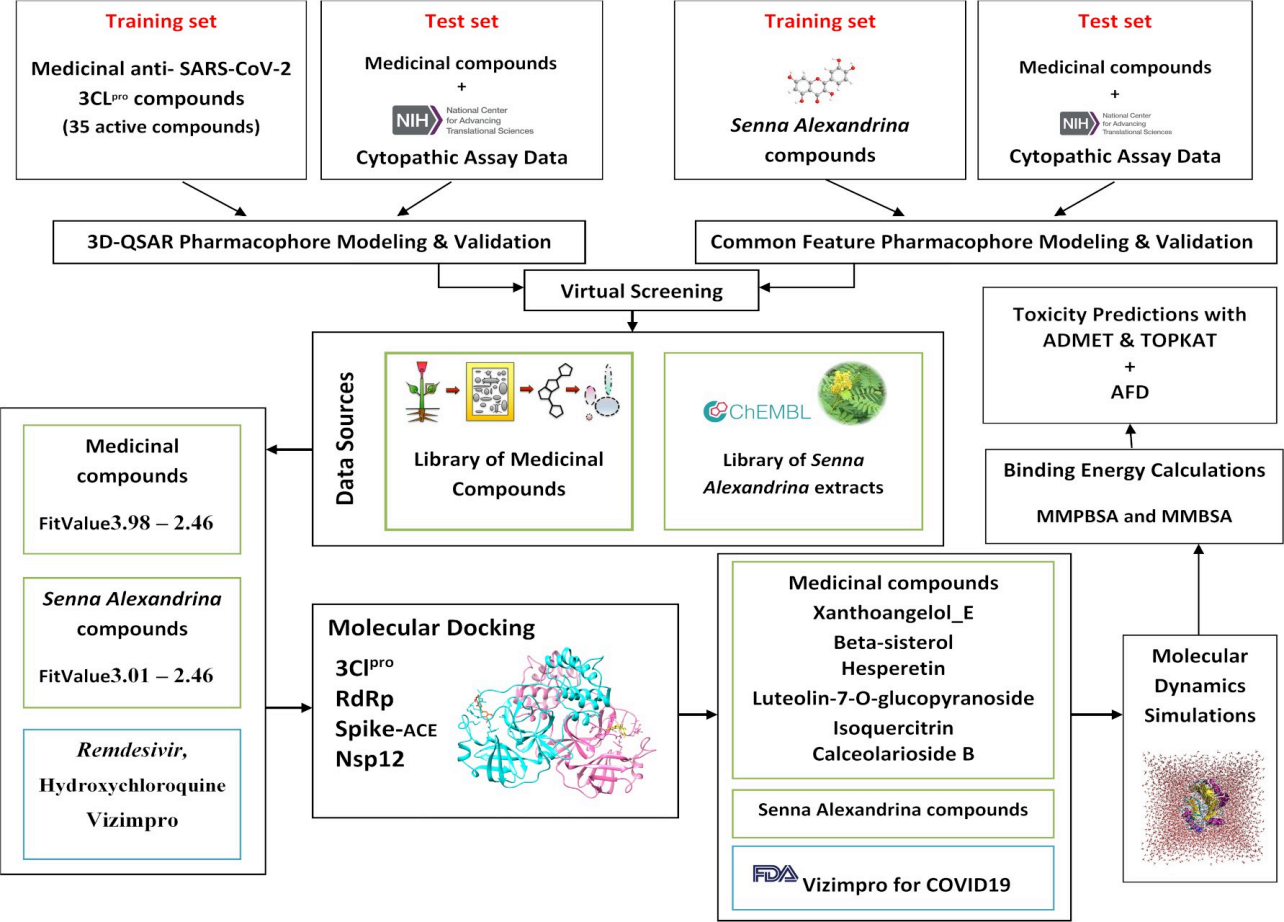
Workflow of the current study to identify potential phytochemicals against Covid19 drug targets from a list of known and social media acclaimed natural products.

The complete workflow of the current study is mentioned in Fig 1.

### Dataset collection for pharmacophore modeling

#### Training set

Two training sets were required for the generation of two pharmacophores namely; 3D-QSAR pharmacophore and common feature pharmacophore. A dataset consisting of diverse structural information with *in vitro* data on medicinal plants that have exhibited high potential in inhibiting 3CL^pro^ was collected from different resources and used for the generation of 3D-QSAR pharmacophore [21–23]. The inhibitory studies conducted on SARS-CoV-2 reported the IC_50_ value of chloroquine between 1.13 to 5.47 μM [24], thus establishing a threshold to include those medicinal compounds that exhibited IC_50_ value from 0–15 μM. Training set for the generation of 3D-QSAR pharmacophore is mentioned in the S1 Table. Whereas, *Senna* compounds constituting phenolic acids, flavonoids, and coumarins with the highest quantity of benzoic acid as well as anthraquinones were used in the training set to generate a common feature pharmacophore (S2 Table).

#### Test set

To validate the generated pharmacophores, we investigated both the known active and inactive medicinal compounds between IC_50_ values 1.2 μM to 226 μM. Moreover, to randomize the test set, a set of 77 known active and inactive FDA-approved compounds recently released against 3CL^pro^ by the National Center for Advancing Translational Sciences (NCATS) ("SARS-CoV-2 cytopathic effect (CPE)", 2021) [25] were also added in the test set. To keep the study consistent with experimental results, we particularly added the controls reported in multiple *in vitro* studies including hesperetin (8.3 μM), aloe-emodin (132 μM), apigenin (280.8 ± 21.4 μM), luteolin (20.0 ± 2.2 μM), quercetin (23.8 ± 1.9 μM), and remdesivir (5 μM) [26].

### Pharmacophore modeling

All the protocols such as; 3D-QSAR pharmacophore modeling, common feature pharmacophore modeling, ligand pharmacophore mapping, and feature mapping were carried out with the BIOVIA Discovery Studio (DS) [27]. In this study, we generated two pharmacophore models; **1)** 3D-QSAR pharmacophore model for biologically active phytochemicals reported against 3CL^pro^ and **2)** a common feature pharmacophore for *Senna* compounds with no reported experimental activity with a given target. 3D-QSAR pharmacophore generation protocol that is designed to generate predictive pharmacophores based on ligands with known activity against a specific biological target was run with input ligands set to a training set of 27 active phytochemicals against 3CL^pro^. The HypoGen algorithm generated 200 conformations with an energy threshold for each ligand maintained within a 10 kcal/mol energy range that were further optimized using simulated annealing. The ligands in the dataset used pre-defined ‘Activ’ and ‘Uncert’ values. ‘Activ’ refers to the tested biological values of the input ligands (IC_50_). Whereas; ‘Uncert’ that is the uncertainty value was set to 1.5 implying the variation between experimental and estimated values during model generation up to two times and also affects the regression fitting of the pharmacophores during optimization. Moreover, to determine the best features that should be considered during the pharmacophore models generation, we used a Feature mapping tool that maps solvent-accessible features and identifies all possible locations of the selected pharmacophore features on the given ligand. The parameters for “Max” and “Min” in the feature mapping protocol were set to 5 and 1, respectively. The results exhibited hydrogen bond acceptor (HBA), hydrogen bond donor (HBD), hydrophobic (HYD), positive ionizable (PI), and ring aromatic (RA) features as commonly mapped features from the training set compounds.

However, in the absence of biologically predicted values for *Senna* compounds against 3CL^pro^, a common feature pharmacophore was generated with the phytochemicals of *Senna* presented in S2 Table. We assumed all compounds to be equally active that were considered as reference ligands exhibiting HBA, HBD, HYD, PI, and RA as common features as a result of the feature mapping tool analysis. The protocol carried out both the model generation with a training set and validation with a test set simultaneously. The output file lists the SD file for each pharmacophore aligned to the ligands and the receiver operating characteristic (ROC) curve exhibiting the trade-off between sensitivity and specificity of selected pharmacophores. Each pharmacophore uses the FitValue property to ascertain which ligand uses the maximum features for mapping. Higher FitValue is indicative of more features mapped in a suitable conformation.

### Pharmacophore validation

Two methods: the cost analysis and test set analysis were used to determine the best 3D-QSAR pharmacophore models. A test set of ligands with similar receptor binding that was not used for model generation was employed to assess the ability of generated pharmacophores to estimate activity of test ligands. The protocol to generate a 3D-QSAR pharmacophore model for the given training set and validation with a test set takes place simultaneously in BIOVIA DS resulting in a detailed report with 10 hypothesis models generated with different statistical parameters. The output file contains an SD file of the input ligands aligned to each pharmacophore with a report summary that includes regression statistics and a plot of LogEstimate vs. LogActiv for the test ligands to quickly determine the best pharmacophore with the ability to predict the activity of test ligands. Whereas; for the cost analysis, the overall cost of each hypothesis was calculated by summing these 3 cost factors; **1)** FitValue property that indicates the total number of features mapped that are HBA, HBD, HYD, PI, and RA **2)** high correlation coefficient (r2), **3)** lowest total cost while exhibiting highest cost difference and **4)** low root mean square deviation (RMSD) values. Another method ligand pharmacophore mapping protocol was run with the test ligands as input to validate the performance of selected pharmacophores and return an estimated value that should be close to the experimental values. It anticipates the activity of selected pharmacophore based on a decent correlation coefficient with the test set ligands and 95% cross-validation confidence.

However, to determine the best common pharmacophore model and to quickly identify the matches, a ligand profiler protocol was used with input data set to test set that was mapped against the generated common feature pharmacophores. Ligand profiler generates a heat map to quickly determine the best set of ligands mapped against a pharmacophore based on the FitValues. From the heat map, the pharmacophores that do not map to any of the test set compounds can be easily identified, making the selection of pharmacophores easier with higher FitValue and more relevant alignment. The validated pharmacophores were subsequently used for lead identification from the molecular libraries.

### Virtual screening for lead identification

The selected 3D-QSAR pharmacophore model was used to screen a library of 2,287 compounds comprising alkylated chalcones, phlorotannins, tanshinones, bioflavonoids, and flavonoids. Whereas, the common feature pharmacophore model was used to screen a library of in-house built *Senna* compounds collected using the Chembl similarity searching tool [28] against sennosides and anthraquinones present in *Senna*. We chose 3D-QSAR pharmacophore model Hypo1 and the common feature pharmacophore model 10 as a 3D query to screen the input ligands against each pharmacophore feature present in the query to extract the more relevant pharmacophore models. The screen library protocol in BIOVIA DS was used with the minimum features parameter set to 3, the maximum features parameter set to 4, and the maximum subset of pharmacophore parameter set to 100. This suggests that 100 pharmacophore subsets of all possible 3 and 4 feature pharmacophore from the 5 features will be used for screening. The resulting subset of both the libraries was then subjected to molecular docking.

### Molecular docking

LibDock algorithm of BIOVIA DS was run to dock the screened ligands from 3D-QSAR pharmacophore of phytochemicals and common feature pharmacophore consisting of *Senna* compounds separately. The screened compounds against 3D-QSAR pharmacophore were reported specifically as anti-3CL^pro^ ligands with known biological activity, thus they were not tested for other Covid19 targets and subjected to docking into the active site His164 of 3CL^pro^ [29]. LibDock calculates a hotspot map for the given receptor site containing polar and apolar groups, which are used to generate favorable ligand-receptor interactions followed by the energy minimization step. Due to the conformational differences in the monomeric and dimeric structure of 3CL^pro^ as suggested in the literature [30], we subjected all the complexes to MD simulations with the monomer; whereas, only those compounds were tested with dimer that were unstable with monomer. Moreover, due to the absence of any experimental data on screened compounds sennoside A, B, C, and D of *Senna* against 3CL^pro^, we expanded our research scope and chose four additional proteins that are crucial for the survival of SARS-CoV-2 namely; spike protein (PDB: 6LZG), helicase nsp13 (PDB: 6JYT), RdRp nsp12 (PDB: 6M71) and 3CL^pro^ (PDB: 6LU7). In order to validate further, based on dynamics and conformational changes, we selected three FDA-approved drugs against Covid19 namely; remdesivir, hydroxychloroquine, and vizimpro [31], which were used as a control. The top-scoring compounds from each dataset were then subjected to molecular dynamics (MD) simulations with Amber16 [32].

### Molecular dynamics simulations

MD simulations were carried out on the active phytochemicals, vizimpro and *Senna* compounds to check the behavior of protein with their proposed ligands. There were 6 phytochemicals and vizimpro in complex with 3CL^pro^, 2 complexes of spike protein (docked at both the regular and allosteric site), helicase nsp13, RdRp nsp12, and 3CL^pro^ with *Senna* compounds. For this purpose, parameter/topology files were generated using the LEaP program followed by the system preparation by neutralizing it with counter ions (Na^+^/Cl^-^). TIP3P tetrahedral solvation box was adjusted for 12 Å and General Amber Force Field (GAFF) and ff14SB was used to get insights into the intermolecular and intramolecular interactions. Furthermore, in the next step, preprocessing was performed for all the systems with 500 steps and 1000 cycles of minimization with 200kcal/mol/Å^2^. The whole system’s atoms were again minimized for 1000 cycles with 5kcal/mol/Å^2^ by applying restraints on carbon alpha atoms, while 300 minimization steps were run for non-heavy atoms to further relax the system. The system was slowly heated with restraints on the backbone and restraint weight 5 kcal/mol/Å^2^ using Langevin dynamics till 300 K. The system was then equilibrated for 100 ps to make it stabilized according to the environment. The pressure was sustained through the NPT ensemble allowing the restraint weight of 5kcal/mol/Å^2^. All these systems then underwent production run of minimum 200 ns with a non-bounded cut-off set to 8.0 Å. However, only those systems were extended that exhibited instability in their RMSD trend mentioned in S7 Fig.

### Trajectories analysis

The generated trajectories were analyzed through Amber CPPTRAJ module 16 to observe the stability of the complex. Different parameters such as RMSD, root mean square fluctuations (RMSF), beta-factor (β-factor), and radius of gyration (Rg) were calculated. The generated graphs and trajectories were analyzed using the Visual Molecular Dynamics [33] and Chimera [34].

### Binding free energies

Binding free energies were calculated using the MMPBSA/GBSA package of Amber16 [35], which used the generated trajectories of MD simulations and subjected them to MM(PB/GB)SA.py module [36]. The system works by calculating the energy difference between complex, unaided protein and ligand. The MMPBSA.py module was used to generate the prmtop files of protein, ligand, and complex, which subsequently follow the total binding energies calculation and decomposition binding free energy calculations. The total 450 frames each after every 0.2 seconds were mined from the entire MD trajectories and exposed to MMPBSA calculations. Poisson-Boltzmann (PB) or Generalized-Born (GB) approaches were used to accomplish the analysis. The binding free energy provides information about the significant residues that help to analyze the components that participated in keeping the protein and ligand intact. Furthermore, these binding energies were decomposed into per residue using the MMPBSA.py module of Amber 16.

### Axial frequency distribution

The axial frequency distribution (AFD) was employed to have insights about the positioning of ligand with respect to coordinates. It gives a detailed assessment of binding pattern of ligand by providing insights into distribution of atoms of ligands with respect to the x and y coordinates. To run AFD, the protein atom was chosen as a point of reference, and a 3D histogram was generated. The axial distribution of atoms is represented through the following equation:

$$AFD=\sum_{i=1,j=1}^{k,l}mi,j$$

Where, i and j represent the coordinates of ligand atoms on X and Y plane, respectively, k and l represent the cutoff values on X and Y plane, whereas m_i,j_ is the number of observations for i and j coordinates [37].

### Toxicity analysis

ADMET protocol of BIOVIA DS software was used to calculate the ADMET properties of selected compounds whereas; TOPKAT suite of BIOVIA DS software carried out the toxicity prediction based on built-in and validated rodent models. The parameters such as solubility level, absorption level, blood-brain barrier (BBB) penetration level, plasma protein binding (PPB) prediction with a cutoff score of -2.209, CYP2D6 (cutoff score 0.161), and hepatotoxic prediction using a cutoff value of -4.154 were calculated. Moreover, the TOPKAT calculated toxicity based on validated models present in BIOVIA DS such as; FDA rodent carcinogenicity test, the prediction of tumorigenic dose rate 50 (TD50) of a drug, rat maximum tolerated dose (MTD), test rat oral acute median lethal dose (LD50) of a chemical, prediction of rat chronic lowest observed adverse effect level (LOAEL), ocular and skin irritancy.

### Network pharmacological analysis

The network pharmacological approach was used to decipher the signaling pathways associated with target proteins of proposed phytochemicals against Covid19. Proposed phytochemicals including *Senna* compounds were subjected to http://www.swisstargetprediction.ch/ to identify the drug targets. Whereas, the DisGeNET (http://www.disgenet.org/) disease target prediction analysis platform was used to find the drug targets of Covid19. Drug targets that were common between Covid19 and phytochemicals were selected and merged by Cytoscape3.9.1 software to construct an active ingredient-key targets Covid19 network.

## Results

### Pharmacophore modeling

Ligand-based 3D-QSAR pharmacophore modeling resulted in the generation of 10 hypotheses that aligned to the ligands present in training set. The best hypothesis Hypo1 was selected based on the highest correlation value of 0.74, highest cost difference of 115.21, and the lowest RMSD of 1.39 Å. The statistical values of 10 hypotheses are summarized in Table 1. The highest cost difference values indicate the ability of pharmacophore to predict estimated values with respect to experimental values with 90% significance. The selection of best pharmacophore model was also based on the highest FitValue of 4.36 that is based on the alignment of training set ligands to the pharmacophore. Four common features consisting of 1 HBA, 2 HYD, and 1 RA were observed in Hypo1 as presented in S1 Fig. It was noted that all the features of pharmacophore were mapped to the most active medicinal compound xanthoangelol_E having IC_50_ = 1.2 ± 0.4 μM, depicted in S1 Fig. Moreover, a list of Hypo1 estimated and experimental values with their corresponding error values against medicinal compounds are also given in Table 2. Interestingly, it was also noted that among the four features, only RA was missing from other active phytochemicals endorsing the importance of HBA and HYD features, which might be responsible for the experimental activity.

**Table 1:** Statistical parameters of top 10 3D-QSAR pharmacophore hypotheses generated using HypoGen algorithm.

Hypothesis No.	Total Cost	Cost Difference	RMSD^b^	Correlation	Features^c^
1	125.43	115.2	1.39	0.741	HBA, HP, RA
2	129.24	111.39	1.44	0.729	HBA, HP, RA
3	137.88	102.76	1.52	0.707	HBA, HP, RA
4	141.68	98.961	1.55	0.697	HBA, HP, RA
5	145.10	95.537	1.57	0.691	HBA, HP, RA
6	145.21	95.429	1.58	0.689	HBA, HP, RA
7	146.72	93.917	1.60	0.682	HBD, HP
8	153.04	87.594	1.65	0.667	HBA, HP, RA
9	154.81	85.827	1.68	0.660	HBA, HP, RA
10	157.02	83.620	1.75	0.653	HBD, HP

The null cost and the fixed cost are 240.64 and 109.19, respectively

^a^ Cost difference between the null and the total cost

^**b**^ RMSD, root mean square deviation

^c^ Abbreviation used for features: HBA, hydrogen bond acceptor; HYD, hydrophobic; RA, Ring Aromatic.

**Table 2:** Predicted and experimental IC_50_ values of the training set compounds based on the 3D-QSAR Hypothesis 1 pharmacophore model.

Name	FitValue	Predicted IC_50 _(μM)	Experimental IC_50 _(μM)	Error	Status	Mapping
Xanthoangelol_E	4.36	1.60	1.20	1.40	active	[6 20 12 9]
Hesperetin	3.68	6.60	8.30	1.50	active	[1 23 14 *]
Iguesterin	3.08	8.10	2.70	3.10	active	[2 25 4 *]
Dieckol	3.14	5.70	2.70	2.10	Moderately active	[12 37 33 *]
Tanic acid	2.12	61	3.00	20	moderately active	[11 * * 61]
Psoralidin	3.71	5.30	4.20	1.30	moderately active	[5 23 17 *]
Tomentin	3.00	7.90	5.00	1.60	moderately active	[3 32 21 *]
Pristimerin	3.07	6.70	5.50	1.20	moderately active	[2 33 24 *]
Amentoflavone	3.00	8.00	8.30	-1.0	moderately active	[9 21 * 13]
Tingenone	2.81	12	9.9	1.20	moderately active	[3 25 5 *]
Betulinic acid	3.03	7.30	10	-1.40	moderately active	[1 31 14 *]
Diplacone	3.11	6.1	10	-1.70	moderately active	[6 29 16 *]
Celastrol	2.74	15	10	1.40	moderately active	[4 16 18 *]
Dihydro tanshinone I	2.18	53	14	3.70	moderately active	[* 15 * 1]
Mimulone	3.13	5.80	14	-2.50	moderately active	[5 22 15 *]

*Refers to the missing features.

However, in the case of *Senna* compounds, it was observed that the common feature pharmacophore no. 10 aligned with the training set compounds with highest FitValues exhibiting 3 HBA and 1 RA features as presented in S2 Fig. The compound isoquercetin mapped to the common feature pharmacophore with highest FitValue of 0.975 as displayed in S2 Fig. However, it is noteworthy that the presence of hydrophobic pockets and basic residues in 3CL^pro^ have been reported in the literature [29], highlighting the significance of hydrophobic features, which is missing in the case of common feature pharmacophore.

### Validation of pharmacophore models

**Fig 2:**
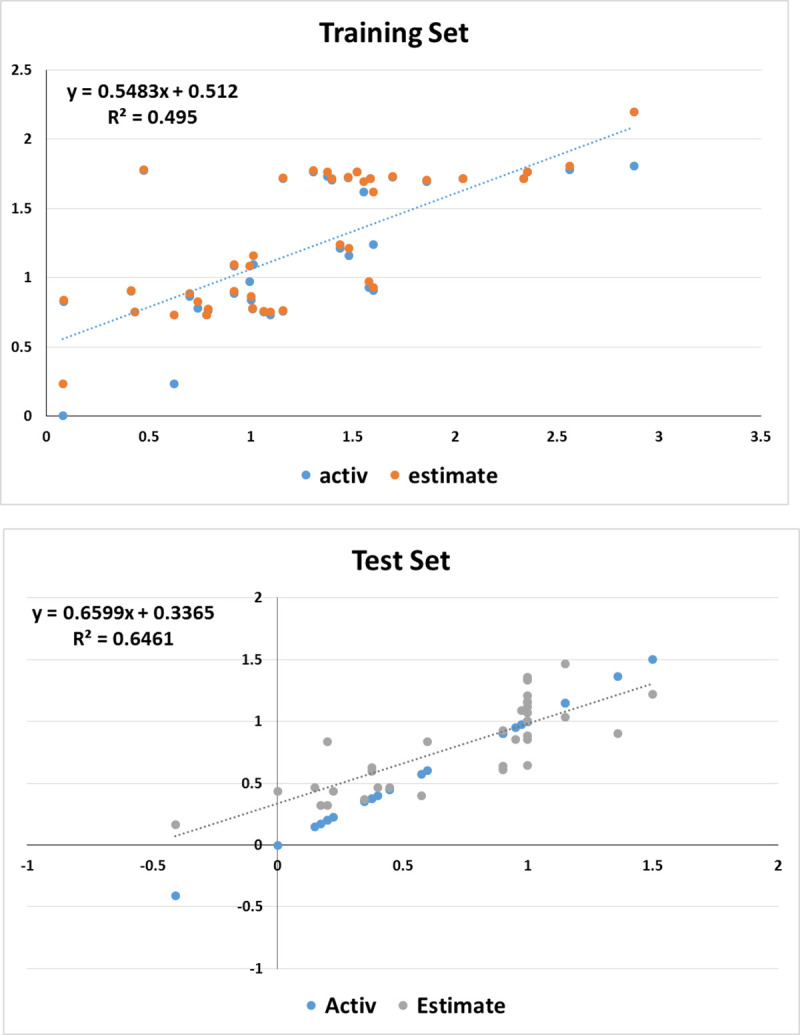
Correlation graph in logarithmic scale for experimental values vs. predicted values of training and test ligands that mapped the 3D-QSAR pharmacophore model Hypo1.

Validation of any 3D-QSAR hypothesis model is primarily based on the cost analysis of two theoretical values **1)** total cost value and **2)** null cost value. A good quality pharmacophore has a cost difference of 40 to 60-bit score representing 70–90% confidence level along with other important parameters such as lowest RMSD and the highest correlation values. However, the 3D-QSAR model selected in this study represents the probability of more than 90% correlation among the data sets with the highest total cost difference 115.21 and highest correlation value 0.74, exhibiting a pharmacophore model with high prediction ability for lead identification. Apart from the cost analysis of the training set, the 3D-QSAR pharmacophore model was validated using error values estimated between the experimental and estimated activity values generated as a result of the ligand pharmacophore mapping protocol. 33 test set compounds mapped to Hypo1 pharmacophore with error value of = <10 depicting order one difference between the experimental and estimated IC^50^ values. The statistical analysis indicated higher regression coefficient (R^2^) value of 0.646 for the test set while cross-validating the results with training set that displayed regression coefficient value R^2^ = 0.495, depicted in Fig 2.

**Fig 3:**
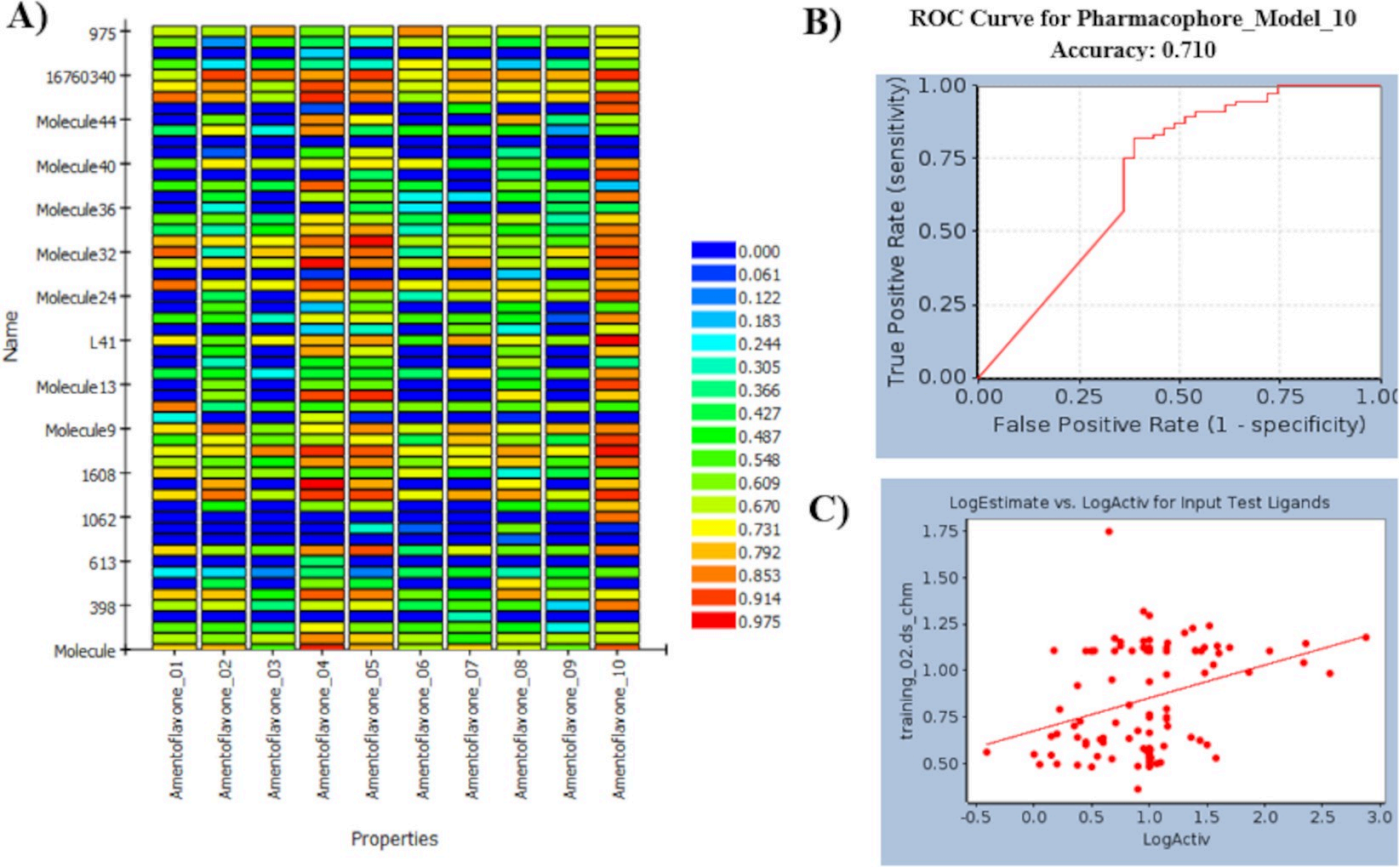
**a)** Heat map generated by the ligand profiler for validation of common feature pharmacophore model No. 10 with the test set **b)** ROC curves generated for the validation of common feature pharmacophore model with the test set **c)** Logarithmic graph between estimated and experimental values of the training set and test set of common feature pharmacophore.

Moreover, the FDA-approved drugs, vilazodone and lapatinib both having IC_50_ of 10 μM from the test set mapped the 3D-QSAR pharmacophore model highest FitValue of 3.56 as depicted in S1 Fig. From these results, it was conceived that the presence of chemical features HBA, HYD, and RA for 3CL^pro^ inhibitory activity are crucial and are also found in FDA-approved drugs currently under clinical trials. Furthermore, in the case of common feature pharmacophore modeled for the *Senna* compounds, the ROC curve was used to evaluate the degree of false positivity of the model with known active and inactive compounds. The curve plots true positives against false positives and indicates if the model predicted active compounds higher than the inactive compounds. The AUC value lies between 0 and 1; 0 is indicative of a bad classifier, however, the selected model exhibited a fair accuracy score of 0.710 as presented in Fig 3. S3 Table displays sensitivity and specificity values of the selected model with an ability to distinguish between active and inactive compounds. Additionally, the results of ligand profiler protocol exhibited mapping of 15 test set compounds accurately with the selected pharmacophore and labeled active compounds as red and inactive as blue that can be seen in Fig 3. It is therefore determined from the validation results that both pharmacophore models exhibited the capability to classify active and inactive compounds. The 3D-QSAR pharmacophore model however exhibited all the significant features that are required for binding of 3CL^pro^, while the common feature pharmacophore model failed to identify hydrophobicity as a common feature.

### Virtual screening

The validated 3D-QSAR Hypo1 model and the common feature pharmacophore model No. 10 were used as a query to screen for new lead compounds from the medicinal library of 2,287 compounds and *Senna* compounds. The compounds from the virtual screening that best mapped to the pharmacophore models were selected based on the highest FitValues. The compounds with FitValue ranging from 3.98–2.46 from each library were selected and subjected to molecular docking.

### Molecular docking

**Fig 4:**
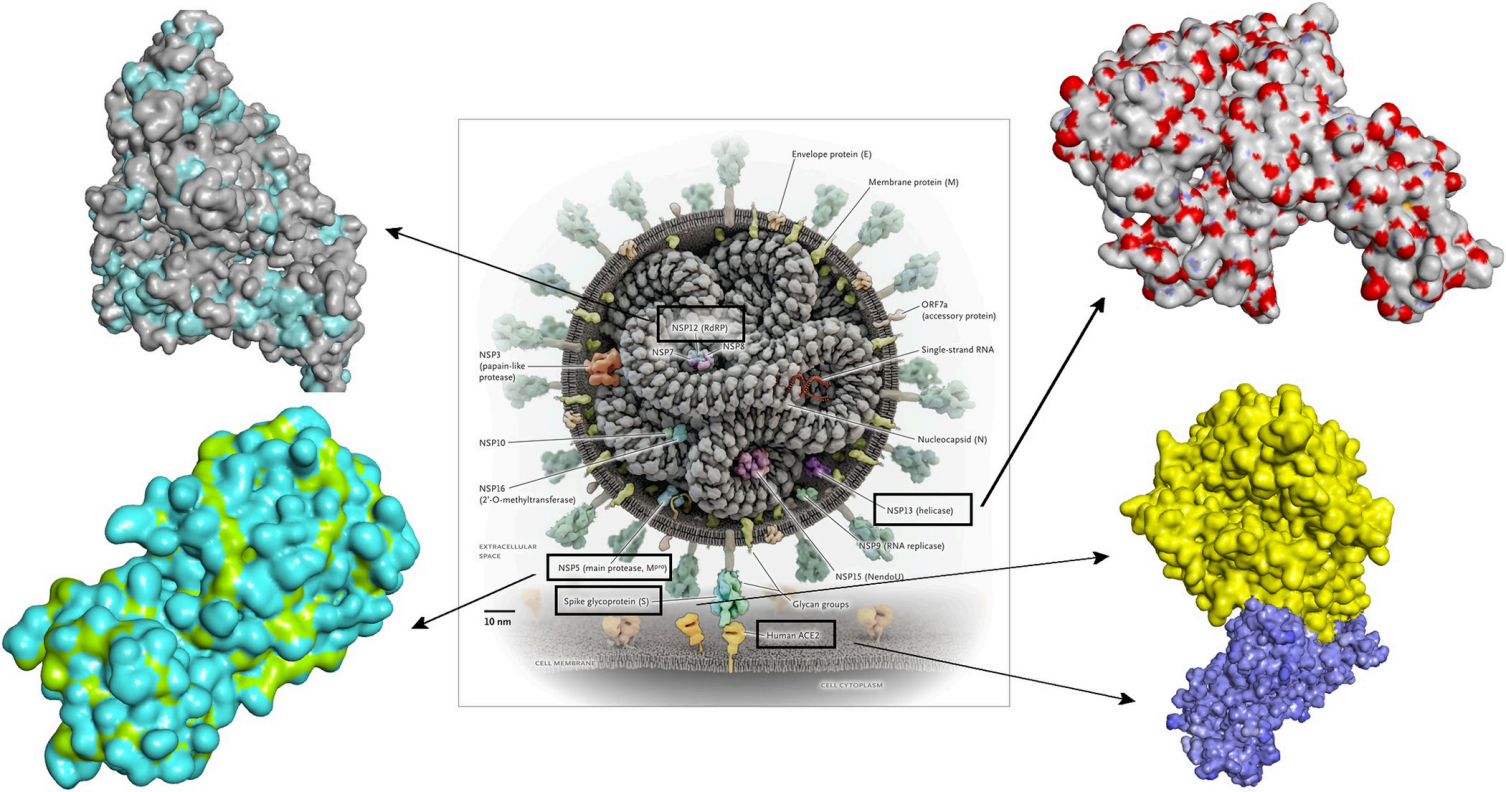
SARS-CoV-2 virion with the four selected proteins. The structure of virion is taken from [40].

Since 3D-QSAR pharmacophore was generated with respect to only one target, the resulting compounds from virtual screening along with the 27 training set compounds of the active phytochemicals were subjected to molecular docking with 3CL^pro^. The top six compounds; xanthoangelol_E with the highest LibDock score of 202, beta-sitosterol with a score of 198, hesperetin with a score of 152, luteolin-7-O-glucopyranoside, isoquercetin, and calceolarioside_B with LibDock score 124, 114, and 112 from the medicinal library were chosen, respectively. However, considering the fact that theoretically one of the tea ingredients of *Senna* may bind a different Covid19 protein and/or key cellular protein, it was necessary to expand our research to determine the binding potential of *Senna* compounds. Therefore, the *Senna* compounds screened for common feature pharmacophore that does not require a specific target for model building were subjected to docking with four additional proteins (both structural and non-structural) of Covid19. The standing of selected proteins in the structure of Covid19 virion is presented in Fig 4. The docked compounds present in high quantity in *Senna* namely; sennoside A, B, C, and D were chosen for further analysis on the basis of Libdock score against each target protein. Herein; we also subjected three recently approved FDA drugs remdesivir, hydroxychloroquine, and vizimpro to molecular docking to validate results based on comparative analysis. However, the topmost compound vizimpro with the highest docking score of 129 was further subjected to MD simulations to further validate representative pharmacophore models and demonstrate their efficiency.

### Active phytochemicals

The topmost compound, xanthoangelol_E is an alkylated chalcone capable of inhibiting both 3CL^pro^ and PL^pro^ in a significant dose-dependent manner with IC_50_ ranging from 1.2 ± 0.4 to 46.4 ± 7.8 μM [38]. Xanthoangelol_E completely occupied the catalytic pocket of 3CL^pro^ making interactions with all the sub-pockets S1, S2, S3, S4, and S5 as depicted in S3 Fig. Whereas, the second and third topmost compounds; beta-sitosterol, and hesperetin, are phytochemicals with reported inhibitory activity for 3CL^pro^ with IC_50_ 115 μM and 8.3 μM, respectively [39]. Both these compounds exhibited strong hydrogen bond interactions with significant residues of 3CL^pro^ such as Glu166, Thr190 and His164 presented in S3 Fig while occupying the sub-pockets S3, S4 and S5. Beta-sitosterol also exhibited other significant interactions such as alkyl, pi-alkyl, and pi-pi T-shaped with residues Leu50, Leu167, Ala191, Met165, Pro168, and His41, respectively. Whereas, hesperetin not only exhibited interactions with the catalytic dyad but the–OH substitutes displayed strong hydrogen bond interactions with Arg489, Gln493, His464, and Leu442.

Furthermore, the fourth topmost compound is luteolin-7-O-glucopyranoside, which is a phytochemical essentially a derivative of luteolin and cynaroside. Luteolin is commonly known for its anti-inflammatory activities both *in vitro* and *in vivo* based on its pharmacologically competent mechanism of action [41]. The glucopyranoside ring of this compound targeted the sub-pockets S1 and S3 while exhibiting interactions with His163, Glu166, and Gln192 as depicted in S4 Fig lying near the catalytic dyad (Cys145- His41). Whereas, the fifth compound, a hydroxycinnamic acid, calceolarioside_B exhibited potential binding affinity with the sub-pockets S1, S2, and S3. The oxygen atoms of the ligand molecule have shown conventional hydrogen bond interactions with Asn142, His41, and Gly143 residues of the binding pocket depicted in S4 Fig. Moreover, the last topmost docked compound, isoquercetin, which is a secondary metabolite from a class of flavonoids, exhibited hydrogen bonding between the aromatic ring of quercetin and the neighboring residues Gln189, Gln192, Val186, and Arg188 as depicted in S4 Fig. Our analysis identified 6 significant active phytochemicals that exhibited strong binding interactions with 3CL^pro^ catalytic triad Cys145-His41 and Glu166. However, the role of Glu166 and the neighboring residues in anchoring the sub-pockets of active site and dimerization of 3CL^pro^ has been regarded as critical by [42, 43], which is analyzed during MD simulation studies in detail.

### *Senna* compounds

To get insights into the binding potential of S*enna* compounds, they were subjected to docking with four additional essential proteins of SARS-CoV-2 to conclude and convey the larger significance of this study.

**Fig 5:**
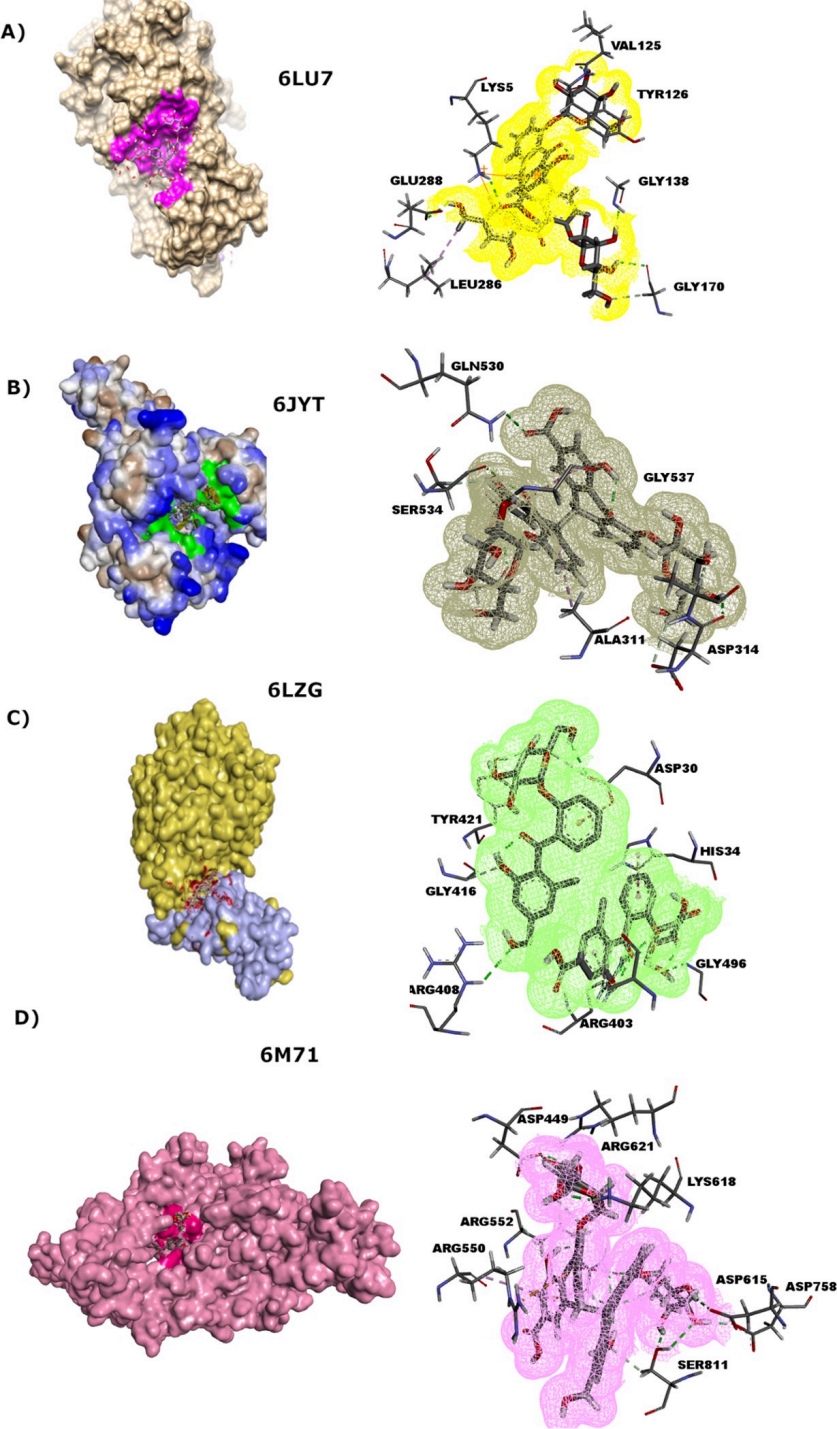
Preferred binding modes of sennosides docked with four essential proteins of SARS-CoV-2 depicting 2D binding interactions. **a)** 3CL^pro^ **b)** Helicase nsp13 **c)** Spike protein-ACE2 receptor complex docked at two different sites **d)** RdRp nsp12.

*3CL*^*pro*^. The docking results of 3CL^pro^ with *Senna* compounds presented in Fig 5 exhibit conventional hydrogen bond interactions with residues Gly170, Gly138, Lys5, Val125, and Glu288 with Libdock score of 109. Compared to the docking results of active phytochemicals against 3CL^pro^, *Senna* has shown weak binding affinity and few intermolecular interactions suggesting that this might not be a potential lead compound for the inhibition of target protein.

*Helicase Nsp13*. Nsp13 is an interesting drug target as it plays a crucial role in the transcription-replication complex of CoVs. Docking of *Senna* compounds against helicase nsp13 has resulted in the Libdock score of 114. Significant hydrogen bond interactions of oxygen atoms of the ligand can be observed in Fig 5 with residues Glu377, Ser312, Gln539, Ser541, Thr288, Arg445, and Glu321. Furthermore, Ala314 and Ala318 were involved in pi-alkyl interactions with benzene ring of the ligand playing a crucial role in adjusting and anchoring the ligand within the binding pocket.

*RdRp Nsp12*. Non-structural proteins such as nsp12 are highly conserved proteins, which are essential for viral infection. *Senna* compounds have shown a Libdock score of 102 against RdRp nsp12. Active pocket residues Trp617, Lys798, Asp760, Cys622, Lys551, and Lys621 have made crucial hydrogen bond interactions with the oxygen atoms of the ligand as depicted in Fig 5. Furthermore, the benzene ring of the ligand has made pi-anion interactions with Asp618.

*Spike protein*. Spike protein aids in the attachment of virus to the host cell and thus is a significant drug target for inhibition studies. Docking of *Senna* compounds against receptor-binding domain (RBD) of spike protein bound with ACE2 receptor resulted in Libdock score of 118. The active site residues involved in crucial interactions with the protein are Asp30, Gly416, Arg408, His34, Ser494, Tyr495, Glu37, Ala387, and Tyr421. Significant hydrogen bond interactions of binding site residues with ligand oxygen atoms have firmly anchored the ligand within the active pocket as depicted in Fig 5.

Furthermore, several other intermolecular interactions such as pi-anion and pi-alkyl between receptor and ligand have also contributed to enhanced binding affinity. Moreover, to analyse the conformational changes in spike-ACE2 complex and to estimate the capability of *Senna* compounds to disrupt interactions between spike protein and ACE2 receptor, molecular docking at the allosteric site of spike protein has revealed a Libdock score of 126. Protein residues Ala330, His356, Glu357, Asp332, and Asn376 have exhibited strong hydrogen bonding interactions with the lead compound. However, Glu384 has exhibited pi-anion interactions with two benzene rings of the ligand.

### Molecular dynamics simulations

All the complexes including vizimpro (the control) were subjected to MD simulations to have insights into the dynamics and conformational stability of active phytochemicals in complex with the SARS-CoV-2 3CL^pro^ and *Senna* compounds with 3CL^pro^, RdRp nsp12, helicase nsp13, and spike-ACE2.

#### Analysis of active phytochemicals

**Fig 6:**
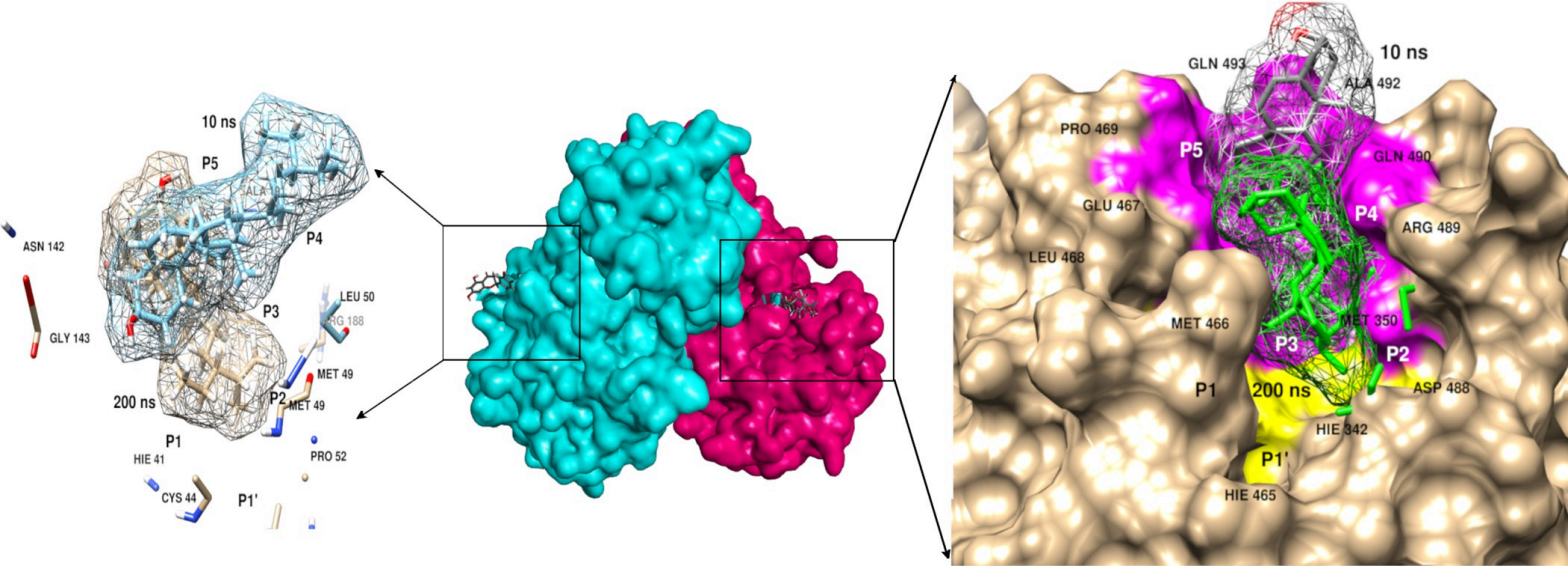
MD simulations of beta-sitosterol exhibited strong electrostatic interactions and stable conformational dynamics with both the chains of dimer 3CL^pro^.

The first compound beta-sitosterol exhibited noteworthy results during 200 ns of MD simulations. It possess stability with both the chains A and B throughout simulations and moved even deeper in the binding pocket of 3CL^pro^ as displayed in Fig 6. Interestingly, beta-sitosterol was observed making alkyl and pi-alkyl interactions more often with the residues of the active site with an average RMSD of 1.78 Å, Rg 22.2 Å and β-factor 31.6 Å as exhibited in S5 Fig. The influence of electrostatic interactions in attaining conformational stability has been explained in the literature several times [44], proposing beta-sitosterol as an interesting candidate for further analysis.

**Fig 7:**
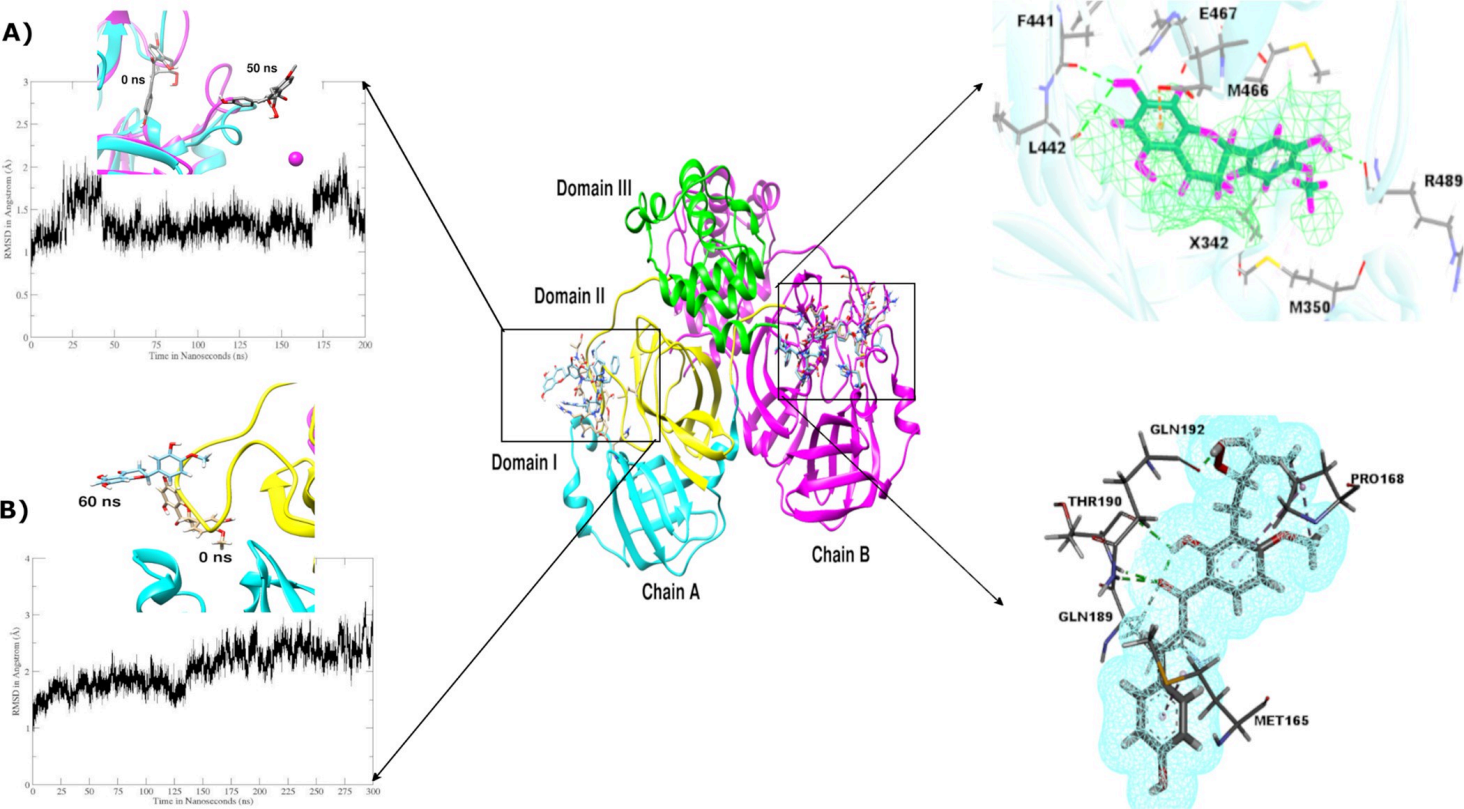
**a)** Hesperetin detached itself from chain A due to structural dynamics in its cavity but stayed intact with chain B till 200 ns as displayed in the top-right image. **b)** Xanthoangelol_E exhibited the same behavior with the chain while displaying strong hydrogen bond interactions and ligand movement deeper in the cavity of chain B till 300 ns, as presented in the bottom-right corner image.

Whereas, the second compound hesperetin was run for 200 ns and exhibited stability for the first 60 ns followed by a sudden increase in RMSD resulting in detachment of hesperetin from chain A, while remaining intact with chain B of the dimer as depicted in Fig 7. Average RMSD of 1.35 Å is shown in S5 Fig, which also exhibits the maximum fluctuations observed mainly in domain III with the Rg value 22.1 Å and β-factor 25.8 Å. Nonetheless, the role of critical residues such as His143, Phe140, Gln189, and Glu166 was again highlighted as they were involved in making significant interactions with chain B of 3CL^pro^.

Similarly, xanthoangelol_E, the most active medicinal compound reported in experimental assays against 3CL^pro^ exhibited the same behavior as hesperetin. However, increase in trend of RMSD graph was observed till 200 ns, which was extended to 300 ns to corroborate the structural dynamics (see S7 Fig for extended simulations). The ligand stayed intact with only chain B of the dimer protein displaying overall stability in protein structure as shown in Fig 7. However, the active site residues of chain B were actively involved in making interactions with xanthoangelol_E with an average RMSD of 2.04 Å as displayed in S5 Fig.

**Fig 8:**
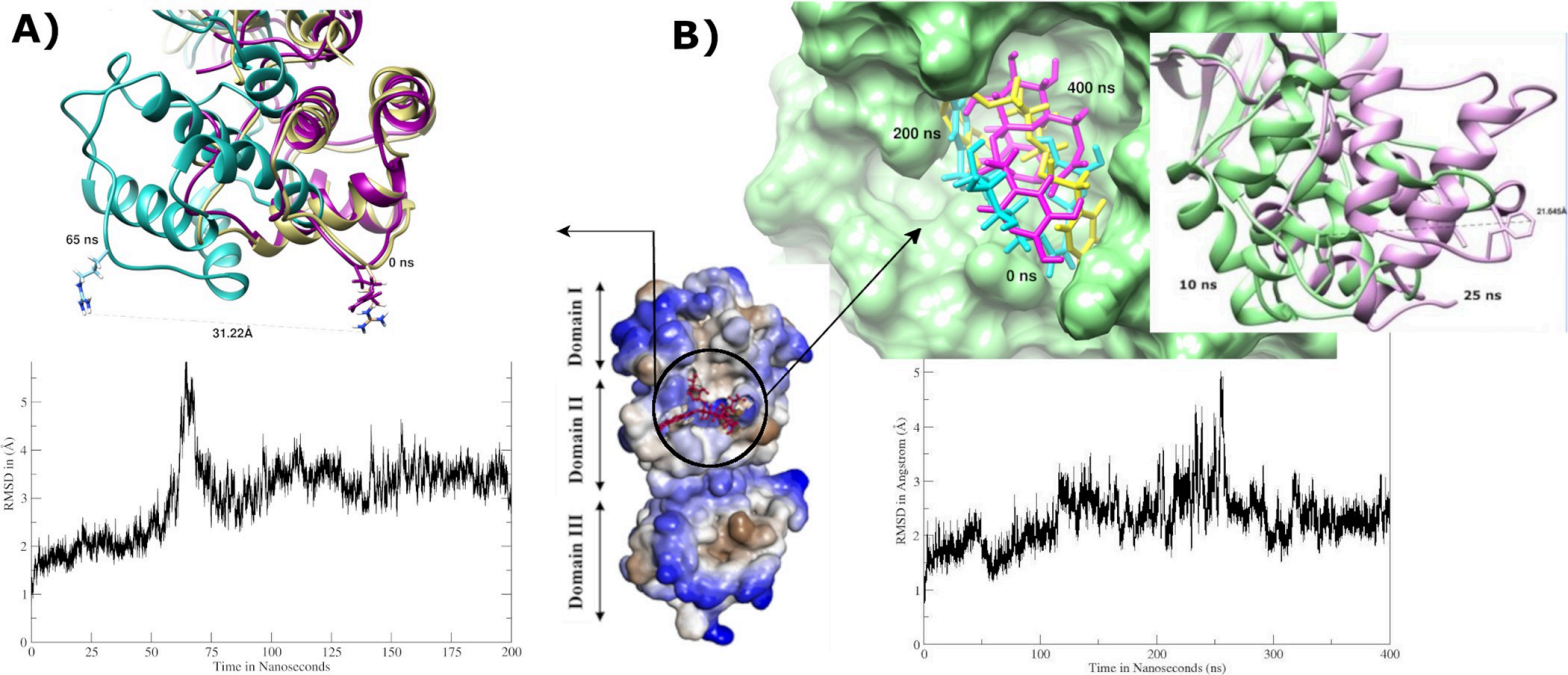
**a)** MD simulations analysis of luteolin-7-O-glucopyranoside for 200 ns. Snapshots from 0 ns, 60 ns, and 200 ns are superimposed depicting an increase in RMSD due to the domain movement. **b)** MD simulations analysis of calceolarioside_B depicting ligand movement attempting to completely occupy the binding site thus leading to the increase in RMSD observed as a consequence of domain movement.

Furthermore, the structural and conformational dynamics of luteolin-7-O-glucopyranoside during 200 ns exhibited extraordinary results with an average RMSD of 3.03 Å exhibiting the formation of 13 hydrogen bonds between glucopyranoside ring and the active site residues of 3CL^pro^. A significant increase was observed between 50 ns to 75 ns with the highest peak noted at 65^th^ ns with an RMSD value of 6.07 Å. This noticeable change can be visually observed in simulation trajectories where increased bonding with the loop region (185–200 residues) that connects domain II to domain III caused the third domain to open up as displayed in Fig 8. Moreover, the number of bonds formed by the ligand significantly increased from 9 to 22, of which, 13 were hydrogen bonds while the rest comprised of C-H bonds and pi-alkyl bonds. The protein was seen to open up to incorporate the ligand into its binding pocket completely at 10 ns forming and breaking hydrogen bonds after every 10 ns up to 75 ns. However, after 80 ns, strong hydrogen bonding was observed stabilizing the RMSD as presented in S6 Fig making domain III returned to original position with the aid of residues Thr190 and Ala191 that kept the critical residues of the active site intact.

The next simulation run with isoquercetin, exhibited fluctuations in domain II and domain III resulting in increased RMSD at 150 ns therefore MD simulations were extended to 300 ns to confirm the structural stability of this complex (see S7 Fig for extended simulations). However, after 200 ns, it was observed that isoquercetin detached from the active site resulting in instability of the complex. Furthermore, the last MD simulation run of calceolarioside_B with 3CL^pro^ exhibited significant fluctuations in RMSD throughout the 200 ns run. The simulations were further extended to 400 ns to explore the stability of the compound in its binding pocket (see S7 Fig for extended simulations). Results yielded showed that a significant rise of RMSD at 250 ns is associated with domain III movement covering 21.6 Å from its initial position as depicted in Fig 8. The ligand adjusted itself in the binding pocket of the receptor to form strong hydrogen bond interactions with crucial residues Glu166, which play a central role in making interactions with a catalytic dyad (Cys145 and His41) and is critical for the proteolytic activity of the viral protein [45, 46]. However, further insights into the simulation results show that the domain moved back to its original position after 300 ns leading to stabilization in RMSD. All the 6 compounds were further compared with a control vizimpro based on MD simulation results, which exhibited the exact behavior between chain B (1–302 residues) of vizimpro and the dimer 3CL^pro^. The ligand from chain A drifted away from the active site after 150 ns. Whereas, the ligand tightly attached itself to chain B and completely occupied the active site while moving inside the cavity making even stronger interactions with the active site residues of the receptor. Our analysis was further extended to the calculation of binding free energies of all the 7 complexes using MMPBSA and MMGBSA calculations to conclude our study.

#### Analysis of *Senna* compounds

**Fig 9:**
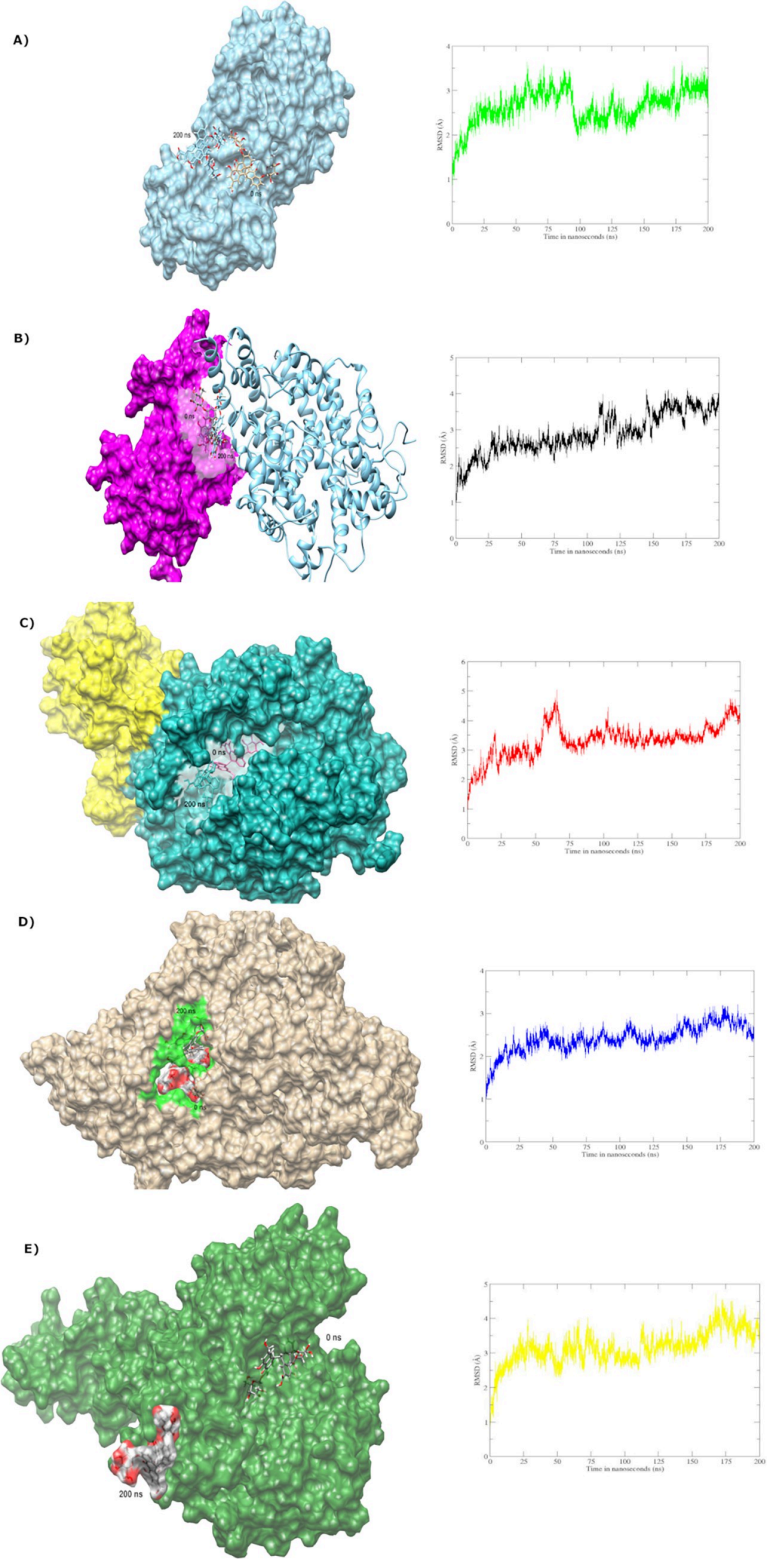
Insights into MD simulations of sennosides with a) 3CL^pro^, b) Spike-ACE2 with ligand attached at RBD, c) Spike-ACE2 with ligand attached at allosteric site, d) RdRp nsp12, and e) Helicase nsp13.

Five complexes were subjected to MD simulations comprising a span of 200 ns each. The first simulation run with 3CL^pro^ exhibited detachment of sennoside (ligand) from the active site as depicted in Fig 9. However, the second most interesting complex with spike protein and ACE2 receptor was analysed with two different sites to identify the conformational changes if any. Ligand bound at RBD of spike protein exhibited stability till 200 ns exhibiting the inability of ligand to abrogate interactions between the spike protein and ACE2 receptor of SARS-Cov-2 as shown in Fig 9. However, the ligand docked at the allosteric site of ACE2 receptor resulted in protein fluctuations but eventually detached itself from the active site after 150 ns as shown in Fig 9. As an outcome, interactions between spike protein and ACE receptor were not abolished that are essential for its inhibitory activity [47, 48]. The same results were observed with RdRp nsp12 and helicase nsp13 complexes. None of the active sites retained sennosides probably due to the absence of hydrophobic interaction, which were reported in different studies as significant for inhibitory activity of SARS-CoV-2 proteins [49, 50]. Moreover, the *Senna* compounds that remained intact with the protein even farther from the active site were still subjected to binding free energy profiles to get insights into the energetics between proteins and ligands; respectively.

### Binding free energy analysis

The end-point free energy calculations were performed to find energetics between active phytochemicals in complex with 3CL^pro^, *Senna* compounds in complex with RdRp and spike-ACE2, and vizimpro. The distribution of energetics of top three active phytochemicals xanthoangelol_E, beta-sitosterol, and hesperetin as a result of MMGBSA calculations is mentioned in Table 3. The sum of binding free energy with a dimer 3D structure of 3CL^pro^ exhibited impressive values ranging from -86.56 to -92.91 kcal/mol. The highest number of binding interactions resulted from the van der Waals interactions with values -163.69 to -172.99 kcal/mol between dimer and the selected ligands. However, the MMGBSA value of vizimpro, a control FDA-approved drug for SARS-CoV-2 exhibited a value of -52.80 kcal/mol. Moreover, the energetics of xanthoangelol_E, beta-sitosterol, and hesperetin as a result of MMPBSA calculations mentioned in Table 3 exhibited values ranging from -22.31 to -26.82 that were striking as compared to the control drug, vizimpro that was -9.56 kcal/mol.

**Table 3:** Binding free energy and its components in MM/GBSA and MM/PBSA for the active phytochemicals, *Senna* compounds, and vizimpro in complex with 3CL^pro^, RdRp, and spike-ACE2 proteins in kcal/mol.

Energy Components	Vizimpro 3CLpro	Beta-Sitosterol 3CLpro	Heperetin 3CLpro	Xanthoangelol,_E 3CLpro	Luteolin 3CLpro	Calceolarioside B 3CLpro	Isoquercetin 3CLpro	Sennoside 3CLpro	Sennoside RdRp	Sennoside Spike-ACE2
**MM/GBSA**										
**VDWAALS**	-52.8713	-172.9954	-163.6940	-170.2654	-62.0288	-45.7566	-38.7973	-23.4553	-47.3276	-34.9620
**EEL**	67.9698	-604.9742	-672.0357	-610.7079	-8.5579	44.2002	123.7085	-15.2853	-133.0586	-34.9601
**EGB**	-61.5635	673.5643	720.7120	671.3829	25.4674	-29.4643	-100.7974	31.6589	147.1077	67.0213
**ESURF**	-6.3440	-22.6008	-21.9985	-22.0860	-7.9486	-5.2809	-4.7543	-2.8773	-7.6417	-4.9811
**DELTA G gas**	15.0985	-737.5308	-791.6253	-738.7731	-70.5867	-1.5564	84.9112	-38.7405	-180.3862	-69.9221
**DELTA G solv**	-67.9075	650.9634	698.7134	649.2969	17.5187	-34.7452	-105.5516	28.7816	139.4660	62.0402
**DELTA TOTAL**	-52.8090	-86.5674	-92.9119	-89.4762	-53.0680	-36.3016	-20.6405	-9.9590	-40.9203	-7.8819
**MM/PBSA**										
**VDWAALS**	-52.8713	-172.9954	-163.6940	-170.2654	-62.0288	-45.7566	-38.7973	-23.4553	-47.3276	-34.9620
**EEL**	67.9698	-604.9742	-672.0357	-610.7079	-8.5579	44.2002	123.7085	-15.2853	-133.0586	-34.9601
**EPB**	-50.0416	595.4107	657.6581	587.1260	39.0509	-22.3992	-85.5626	29.4452	159.2216	63.4209
**ENPOLAR**	-34.9171	-124.0661	-116.7880	-119.7822	-41.3287	-26.2835	-24.6040	-15.9147	-36.8856	-25.2096
**EDISPER**	60.2918	243.8727	223.9269	236.2362	74.5756	50.7137	46.2899	33.8181	72.2494	55.3440
**DELTA G gas**	15.0985	-737.5308	-791.6253	-738.7731	-70.5867	-1.5564	84.9112	-38.7405	-180.3862	-69.9221
**DELTA G solv**	-24.6669	715.2173	764.7971	703.5800	72.2978	2.0309	-63.8767	47.3485	194.5854	93.5553
**DELTA TOTAL**	-9.5684	-22.3135	-26.8282	-35.1931	1.7110	0.4745	21.0345	8.6080	14.1991	23.6332

Whereas, energetics of other three compounds namely; luteolin-7-O-glucopyranoside and calceolarioside B, and isoquercetin exhibited MMGBSA calculations that lied between -20.64 to -53.06i kcal/mol. The major contributions came as a result of van der Waals interactions with values ranging from -38.79 to -62.02 kcal/mol. Moreover, total binding energy values from MMPBSA calculations exhibited values ranging from 0.4745 to 21.0345 kcal/mol with mostly contributions from the van der Waals interactions that lied between -45.75 to -62.02 kcal/mol. Furthermore, free energy calculations of all three complexes with sennosides lied from 8.60 to 23.63 kcal/mol, which were comparatively weak as compared to active phytochemicals and the control drug, vizimpro.

### Axial frequency distribution

**Fig 10:**
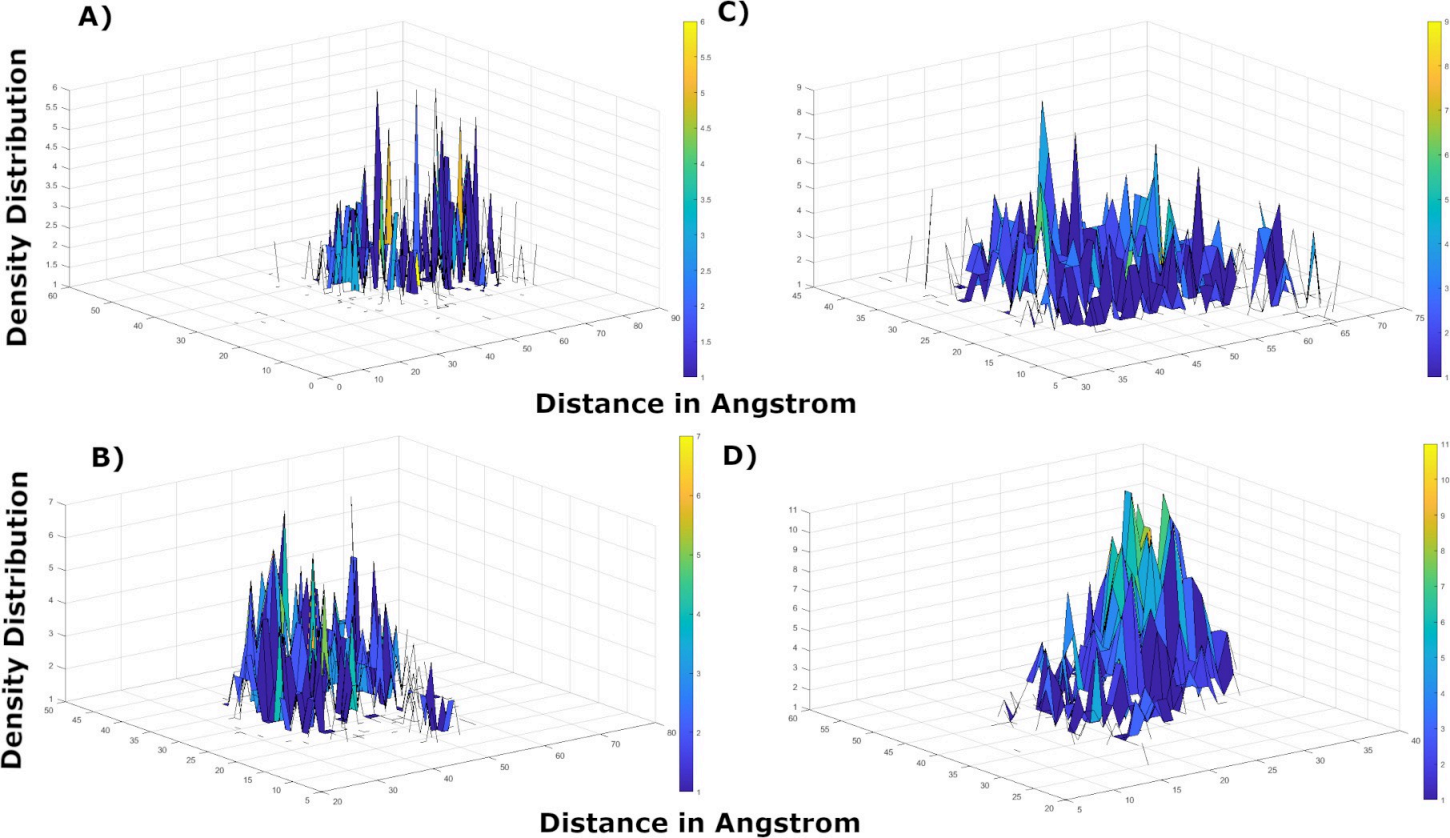
AFD plots to compare point of coordinates and geometry between the active phytochemicals **a)** Hesperetin at 10 ns, **b)** Hesperetin at 200 ns, **c)** Xanthoangelol_E at 10 ns, **d)** Xanthoangelol_E at 200 ns, **e)** Vizimpro at 10 ns, and **f)** Vizimpro at 200 ns.

AFD was carried out on those complexes that exhibited conformational changes/movements in ligand-receptor complex to attain stability during MD simulations. The findings were consistent with MD simulations analysis that exhibited change in orientation of hesperetin while forcing itself to move deeper into the binding cavity while making interactions between His164@NE2 and the ligand atoms O6. Hesperetin displayed higher density distribution with His143 at the beginning of the simulation, which was later shifted to His41 of the second chain covering more surface area but decreased density distribution as exhibited in Fig 10.

Similarly, xanthoangelol_E and vizimpro exhibited weak hydrogen bonding with His residues in the first 10 ns however it displayed higher density distribution later while retaining stable interactions with the same residue. Both the ligands displayed movement around the active site as seen in Fig 10 and exhibited stronger interactions at the end of simulations.

**Fig 11:**
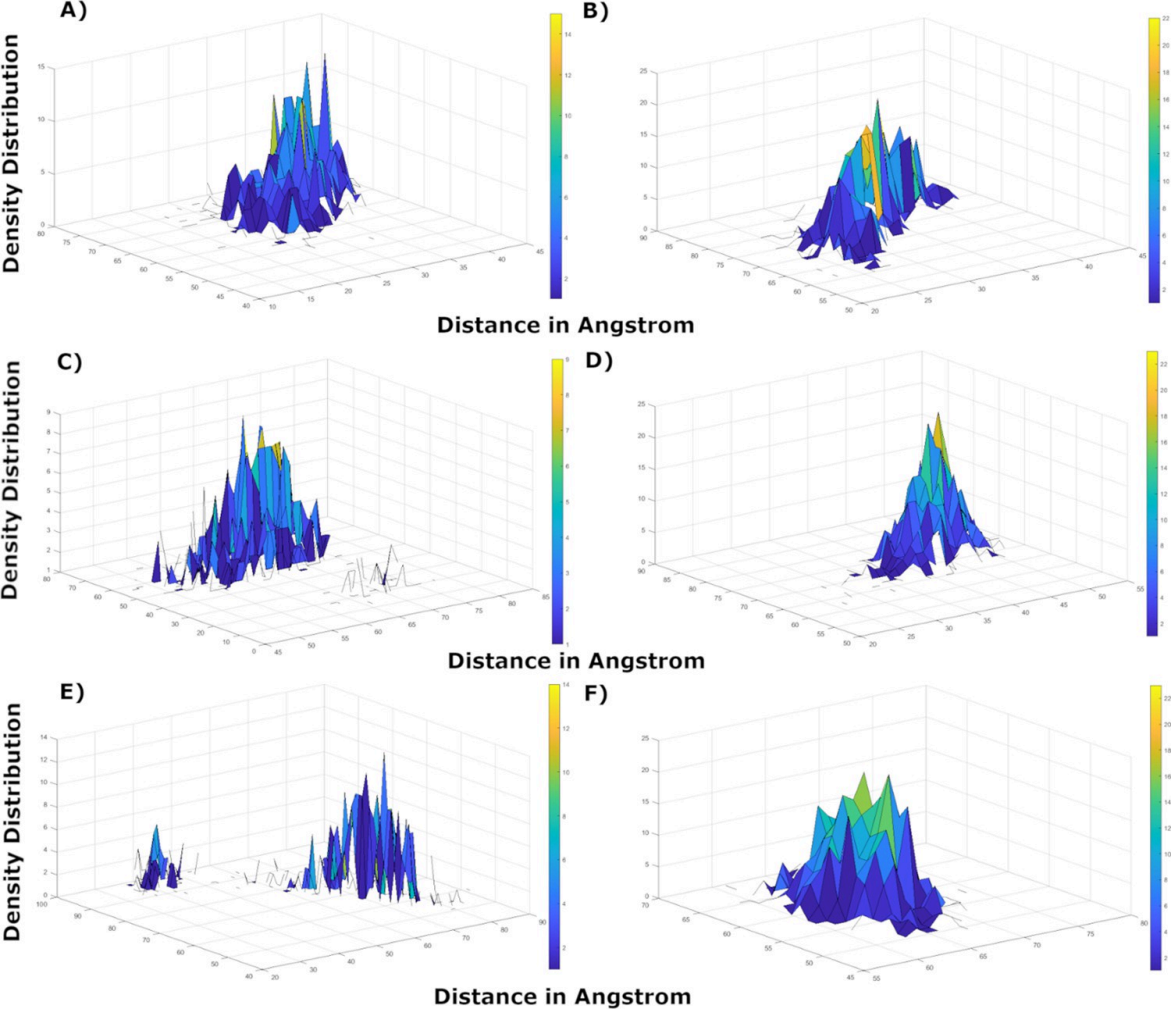
AFD plots to compare point of coordinates and geometry of **a)** Calceolarioside_B at 10 ns, **b)** Calceolarioside_B at 400 ns, **c)** Luteolin-7-O-glucopyranoside at 10 ns, and **d)** Luteolin-7-O-glucopyranoside at 200 ns.

Moreover, luteolin-7-O-glucopyranoside and calceolarioside_B also exhibited hydrogen bonding between two His residues (His41@O of Chain A and His143@HE2 of chain B) and ligand atoms H35 and O13 in the first 10 ns. Fig 11 exhibits instabilities in density distribution between calceolarioside_B atoms and His residues however the fluctuations in distances are representative of ligand movement between the two chains. Whereas, lutelolin exhibited strong hydrogen bonding with maximum density distribution together with decrease in distance between His143 and the ligand atoms till the end of MD simulations (200 ns), presenting luteolin as more stable than calceolarioside_B. The results from AFD highlight the significance of His residues present in both the chains of 3CL^pro^ that participated in maintaining the stability of all the complexes under study.

### Pharmacokinetic profiling analysis

#### ADMET analysis

The ADMET analysis exhibited good absorption values of xanthoangelol_E and hesperetin indicating ability of both the compounds to enter blood circulation as compared to beta-sitosterol, and *Senna* compounds including sennoside A and sennoside B as displayed in Table 4. Notably, only hesperetin presented less blood-brain barrier (BBB) penetration ability to penetrate CNS as compared to other active phytochemicals that displayed high or very high BBB values. Moreover, hesperetin and beta-sitosterol exhibited good solubility values as compared to xanthoangelol_E, and calceolarioside_B. Furthermore, xanthoangelol_E and beta-sitosterol emerged as non-inhibitors of CYP2D6 exhibiting negative hepatotoxicity and greater than 90% ability to bind to plasma proteins.

**Table 4:** ADMET properties of chemical compounds present in *Senna* in comparison with the active phytochemicals and FDA-approved drugs.

Name	^a^Absorption level	^b^BBB level	^c^Solubility Level	^d^CYP2D6	^e^Hepatotoxicity	^f^PPB
**Cynaroside**	3	4	3	FALSE	TRUE	FALSE
**Gallic acid**	0	3	4	FALSE	TRUE	FALSE
**Benzoic acid**	0	3	4	FALSE	TRUE	FALSE
**Kaempferol**	0	3	3	FALSE	TRUE	FALSE
**Isorhamnetin**	0	4	3	FALSE	TRUE	FALSE
**Psoralen**	0	2	3	FALSE	TRUE	FALSE
**Syringic acid**	0	3	4	FALSE	TRUE	TRUE
**Vanillic acid**	0	3	4	FALSE	FALSE	FALSE
**Sennoside A**	3	4	2	FALSE	TRUE	FALSE
**Sennoside B**	3	4	3	FALSE	TRUE	FALSE
**Xanthoangelol_E**	0	4	2	FALSE	FALSE	TRUE
**Beta-sisterol**	3	4	3	FALSE	FALSE	TRUE
**Hesperetin**	0	3	3	TRUE	TRUE	FALSE
**Calceolarioside B**	3	4	1	FALSE	FALSE	FALSE
**Isoquercetin**	3	4	3	FALSE	FALSE	FALSE
**Luteolin**	0	4	3	TRUE	TRUE	FALSE
**Vilazadone**	1	4	2	FALSE	TRUE	FALSE
**Lapatinib**	2	4	3	FALSE	TRUE	TRUE
**XL-888**	1	4	3	FALSE	TRUE	TRUE
**Hydroxychloroquine**	0	1	3	TRUE	TRUE	FALSE
**Remdesivir**	3	4	2	FALSE	TRUE	FALSE

^a^Absorption level: (0, good; 1, moderate; 2, poor; 3, very poor)

^b^Blood brain barrier (BBB) level; (0, very high; 1, = high; 2, medium; 3, low; 4, very low)

^c^Solubility level (0, extremely low; 1, very low; 2, low; 3, good; 4, optimal)

^d^CYP2D6 prediction: (cutoff score 0.161)

^e^Hepatotoxicity prediction: (cutoff score -4.154)

^f^Plasma protein binding (PPB) value: (cutoff score -2.209 presenting >90% binding ability of compounds to the plasma protein.

#### Toxicity analysis

The results of TOPKAT are compared with the FDA-approved drugs for the purpose of protocol validation and comparative analysis given in Table 5. The toxicity analysis suggested two active phytochemicals; xanthoangelol_E, and hesperetin as non-carcinogens and non-mutagen as compared to beta-sitosterol and *Senna* compounds Moreover, the TD50 rat model exhibited values ranging from 148–0.19 mg/Kg^-1^ body weight as compared to remdesivir that displayed TD50 value 0.96 mg/Kg^-1^ body weight. Most of the *Senna* compounds exhibited high TD50 values as compared to hesperetin and beta-sitosterol.

**Table 5:** Toxicity properties of chemical compounds present in *Senna* in comparison with the active phytochemicals and FDA-approved drugs calculated with TOPKAT.

Name	FDA Rodent Carcinogenicity	^a^Carcinogenic potency TD50 Rat	^b^Rat MTD feed	^c^Oral LD50	^d^RAT chronic LOAEL	Ames prediction	Skin irritancy	Ocular irritancy
**Cynaroside**	Non-Carcinogen	12.65	1.23	1.35	0.03	Non-Mutagen	None	Moderate
**Gallic acid**	Carcinogen	101.13	1.85	1.29	0.29	Non-Mutagen	None	Moderate
**Benzoic acid**	Single-Carcinogen	148.56	1.15	1.60	0.28	Non-Mutagen	None	Moderate
**Isorhamnetin**	Non-Carcinogen	11.72	0.69	1.20	0.11	Mutagen	None	Mild
**Kaempferol**	Non-Carcinogen	54.54	1.03	0.95	0.14	Mutagen	None	Moderate
**Psoralen**	Multi-Carcinogen	16.52	0.05	0.27	0.01	Non-Mutagen	Mild	Mild
**Syringic acid**	Non-Carcinogen	47.00	0.40	1.84	0.16	Non-Mutagen	None	Moderate
**Vanillic acid**	Non-Carcinogen	77.02	0.38	2.38	0.19	Non-Mutagen	None	Moderate
**Sennoside A**	Non-Carcinogen	0.37	1.99	11.27	0.08	Non-Mutagen	None	Mild
**Sennoside B**	Non-Carcinogen	0.37	1.99	11.27	0.08	Non-Mutagen	None	Mild
**Xanthoangelol_E**	Non-Carcinogen	131.03	0.46	3.70	0.10	Non-Mutagen	Mild	Mild
**Beta-sisterol**	Single-Carcinogen	0.71	0.03	1.57	0.001	Non-Mutagen	Moderate	None
**Hesperetin**	Non-Carcinogen	8.66	0.45	0.925	0.07	Non-Mutagen	None	Mild
**Calceolarioside B**	Non-Carcinogen	2.17	2.62	5.56	0.05	Non-Mutagen	Mild	Mild
**Isoquercetin**	Non-Carcinogen	4.54	1.75	0.84	0.07	Non-Mutagen	None	Moderate
**Luteolin**	Non-Carcinogen	140.46	0.83	0.77	0.11	Non-Mutagen	None	Mild
**Vilazadone**	Non-Carcinogen	0.93	0.13	1.17	0.02	Non-Mutagen	None	Moderate
**Lapatinib**	Non-Carcinogen	9.62	0.15	2.22	0.01	Non-Mutagen	None	Mild
**XL-888**	Non-Carcinogen	0.24	0.08	0.74	0.02	Non-Mutagen	None	Moderate
**Hydroxychloroquine**	Non-Carcinogen	1.30	0.357	0.20	0.03	Mutagen	None	Severe
**Remdesivir**	Non-Carcinogen	0.96	0.09	0.27	0.001	Non-Mutagen	Mild	None

^a^Tumorigenic dose rate 50 TD50 in unit mg/kg body weight

^b^Maximum tolerated dose MTD in unit g/kg body weight

^c^Median lethal dose LD50 in unit g/kg body weight

^d^Lowest observed adverse effect level LOAEL in unit g/kg body weight.

Moreover, the active phytochemicals exhibited rat MTD values ranging from 2.62–0.05 g/Kg body weight while beta-sitosterol displayed lowest MTD values 0.03 g/Kg body weight. Comparatively, FDA-approved drugs including XL-888, vilazadone, and remdesivir presented MTD values of 0.24, 0.93 and 0.09-g/Kg body weights, respectively. Furthermore, the oral LD50 and rat chronic LOAEL values of active phytochemicals stayed within the range of 11.27–0.62 g/Kg body weight and 0.29–0.001 g/Kg body weight, respectively. However, the LOAEL of beta-sitosterol was lower than other active phytochemicals. Notably, *Senna* compounds came out as ocular irritants but predicted the values of skin irritancy as non-moderate. The statistical data of TOPKAT analysis is exhibited in Table 5.

### Network pharmacological analysis

**Fig 12:**
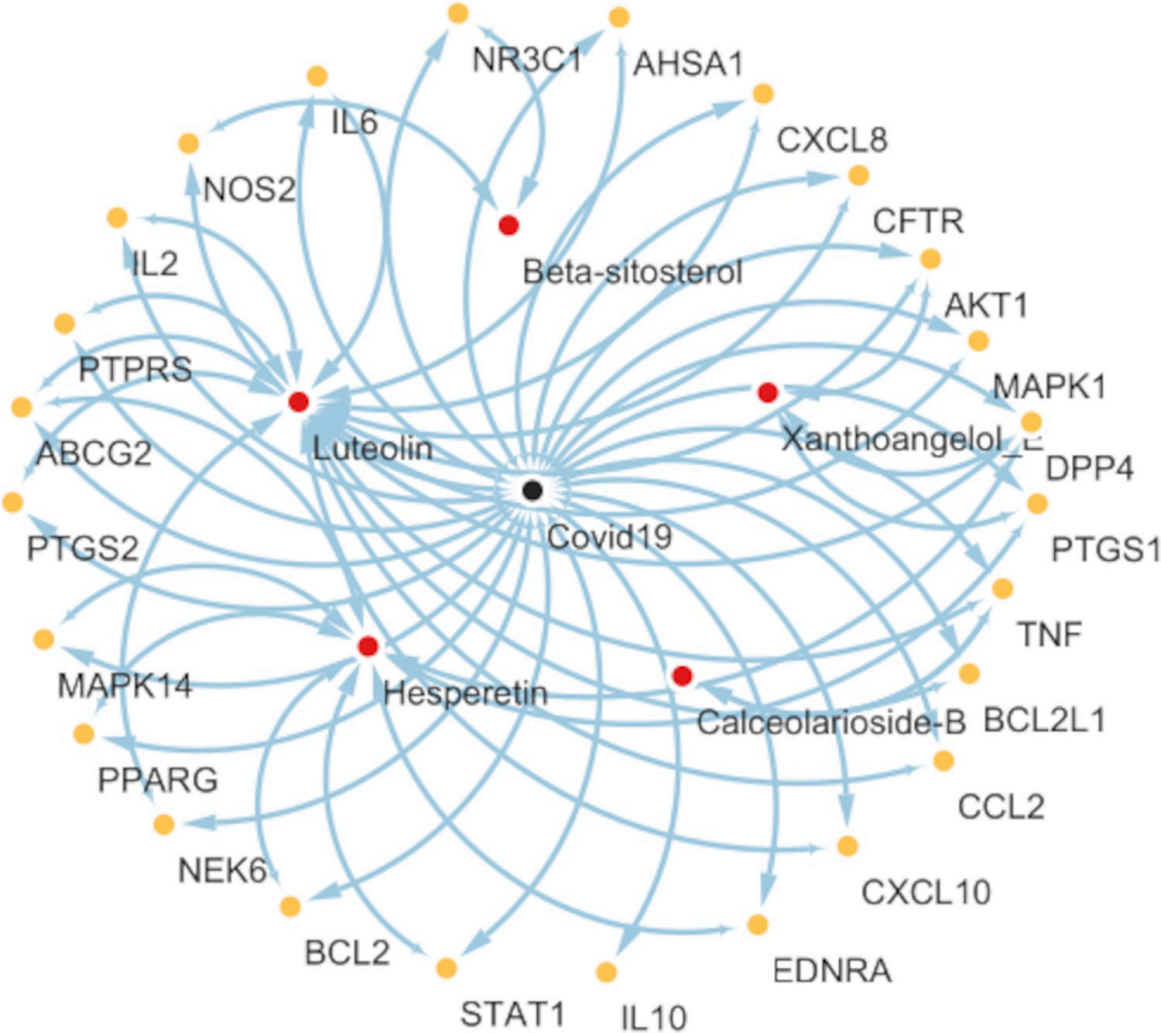
Medicinal compounds disease-target-network. Red dots represent the five-screened active phytochemicals and yellow dots represent the drug targets common between the proposed active phytochemicals and Covid19.

482 Covid19 drug target genes were predicted along with 8 xanthoangelol_E drug target genes, 44 target genes for beta-sitosterol, 88 for hesperetin, 24 for calceolarioside-B, 100 for luteolin, and only 2 for sennosides. Complete details of drug target genes corresponding to phytochemicals are listed in the S4 Table with Uniprot IDs, common names, and target class. Moreover, to generate a network of common interactions between target genes of Covid19 and phytochemicals, only those drug targets were selected that were common between both the groups as presented in Fig 12. Active phytochemicals that were most actively involved in interactions were beta-sitosterol, hesperetin, and luteolin. However, the sennosides failed to exhibit any interactions with Covid19 drug target genes.

The Network pharmacological analysis also highlighted the significance of proposed compounds by identifying common drug targets with Covid19 (presented in Fig 12). The phytochemicals hesperetin, luteolin, and xanthoangelol_E exhibited the maximum number of interactions and identified AKT1, PTGS1, TNF, and DPP4 as the most affected drug targets. Role of these kinases, oxidoreductases, upregulation of DPP4, and inhibition of PI3K/AKT signaling pathway have been highlighted in multiple studies in the treatment of Covid19 [51–53].

## Discussion

While the spread of SARS-CoV-2 is inevitable despite the availability of different vaccines [54], it is imperative to explore every possible treatment to curb this viral disease. Based on viral studies conducted in clinical trials by the U.S. Food and Drug Administration, 2020, the use of combination therapy has been highly recommended such as lopinavir, ritonavir in combination with chloroquine, hydroxychloroquine, and interferon-alpha since they have exhibited potential in the treatment of SARS-CoV-2 infection [55, 56]. Similarly, the combination therapy with natural products has delivered promising results while targeting the specific stages of viral life cycle critical for its survival [57, 58]. It is therefore indispensable to expand the treatment options and include medicinal plants in our research.

In the current study, we derived two pharmacophore models 1) a 3D-QSAR pharmacophore model focused on active phytochemicals that have exhibited biological activity (IC_50_) against 3CL^pro^ with less or no toxicity, and 2) a common feature pharmacophore model derived from *Senna* compounds due to its popularity in India, Pakistan, China, Thailand, Singapore, South East Asia, and inhabitants of these regions residing in the United States, U.K, and Europe as Covid19 treatment. The ligand-based pharmacophore models were validated with FDA compounds in clinical trials for inhibition of main proteases released by the NCATS [25]. 3D-QSAR pharmacophore model identified HBA, HYD, and RA as common features from the training set of active phytochemicals and validated with a test set. Whereas, a common feature pharmacophore model lacked a significant attribute of hydrophobic groups in its framework that is essential for the bioactivity of 3CL^pro^ [47, 48]. Moreover, the 3D-QSAR pharmacophore also mapped to the FDA-approved drugs vilazodone and lapatinib from the test set that are currently in clinical trials for SARS-CoV-2. Vilazodone is a novel anti-depressant and its use in combination with ritonavir and lopinavir that works by affecting the CYS3A4 is reported to inhibit 3CL^pro^ with IC_50_ values below 15 μM [59–61]. Whereas; lapatinib suppressed the SARS-CoV-2 cytopathic effect and cleaved clustering of N protein in MRC5 (human pulmonary fibroblast cell line), thus exhibiting the potential to block 3CL^pro^ [62]. The above results validate the ability of 3D-QSAR pharmacophore to predict the activity of test ligands as active and inactive. Thus, a 3D-QSAR pharmacophore model comprising all the fundamental features for 3CL^pro^ activity proved to be superior to the common feature pharmacophore.

Furthermore, the MD simulations and binding free energy analysis conducted on screened compounds using 3D-QSAR pharmcophore identified xanthoangelol_E, hesperetin and beta-sitosterol as promising inhibitors of 3CL^pro^ exhibiting binding energy values of -35.1 kcal/mol, -26.9 kcal/mol, and -22.3 kcal/mol respectively. Beta-sitosterol displayed hydrophobic interactions with both the chains of a dimer throughout 200 ns probably because of the hydrophobic constituents in its chemical structures. It is noteworthy that xanthoangelol_E and hesperetin displayed higher binding energies than beta-sitosterol even though they stayed intact with only one chain of the dimer. The findings of MD simulations revealed the significance of hydrophobic interactions in keeping the ligands intact with a dimer while it has been previously established both computationally and experimentally that only one chain of dimer 3CL^pro^ is active at a time [30]. In agreement with the simulations studies, experimental analysis conducted by Cheng-Wen Lin *et al*., 2005 [39] on the inhibitory activity of main proteases also revealed xanthoangelol_E as the most active compound against 3CL^pro^ and PL^pro^ with IC_50_ values (11.4 ± 1.4 μM) and (1.2 ± 0.4 μM) respectively [38]. In another study by Park *et al*. 2016, hesperetin and beta-sitosterol were reported to be capable of inhibiting main protease dose-dependently in both the cell-free and cell-based assays with the IC_50_ value of 8.3μM and 115 μM, respectively [39]. Keeping in view the scope of this study and capability of these compounds in experimental assays to inhibit both the 3CL^pro^ and PL^pro^, we also subjected PL^pro^ to molecular docking followed by 50 ns MD simulations on top complexes (S5 and S6 Tables), which can be used as a lead to design more specific dual inhibitors in future (S8 Fig). The structural insights of these compounds might offer clues for development of anti-SARS-CoV-2 drugs and could be used in combination as alternate medicine to prevent Covid19 infection.

In comparison, the secondary metabolites like sennosides A, B, C, and D present in higher percentage in *Senna* [12] exhibited instability and weak binding affinity during computational analysis against 3CL^pro^. Furthermore, to explore the possibility of binding of *Senna* compounds with other Covid19 target proteins, we expanded the prospect of our research. The findings suggested that *Senna* compounds failed to act as a candidate of high ligand affinity capable of disrupting contact between the spike protein RBD and ACE2 receptor. Moreover, these sennoside ligands completely left binding sites of all other targets namely helicase nsp13, spike-ACE2, and 3CL^pro^ except RdRp nsp12 exhibited in Fig 9, which also resulted in displacement of sennoside with weak binding energy (14.199 kcal/mol). The ADMET and TOPKAT protocols have also marked few *Senna* compounds as carcinogens and mutagens after undergoing rat carcinogenicity, TD50, LD50, and LOAEL tests. In conclusion, these findings discredit the use of *Senna* tea as whole with all its chemical constituents in Covid19 treatment, which can rather be harmful in the absence of insufficient clinical data.

However, as an outcome of this work, it is suggested as a matter of general global dissemination that *Senna* tea has nothing to do with the killing of the SARS-CoV-2. This unintentionally held us responsible for deaths of geriatric patients particularly, via diuresis/dehydration/kidney failure through overconsumption of *Senna* tea. Negation of false claim through scientific research is necessary not only to reduce overall burden but at the same time uncertified facts when disseminated through the electronic and print media, is and can remain a curse under the situation of deadly pandemic https://twitter.com/i/status/1262403722006724608. Statements without proper workout from renowned personalities are cherry on top that may have disastrous impact to an already poverty and/or illiteracy hit regions of the world, which is avoidable [63]. While our analysis has a limitation of providing a predictive viewpoint of *Senna* compounds in the absence of experimental data on this plant, further clinical analysis should be considered and implemented in the future. Our study hints and thus recommends that the use of media to propagate any unscientific thought or actions without scientific curation and clarification must be discouraged worldwide. This also urges the need for a scientific body from the WHO platform to control the propagation of scientific information in a manner to prevent a wider community worldwide from spreading unchecked information under the disguise of scientific nomenclature.

## Supporting information

S1 Table Small molecule medicinal inhibitors of 3CL^pro^ with reported activity (IC_50_ values) against SARS-CoV-2 selected as training set for 3D-QSAR pharmacophore modeling. (DOCX)

S2 Table Chemical compounds of *Senna alexandrina* of *Cassia senna* and *Tinnevelly senna* of *Cassia angustifolia* used for common feature pharmacophore modeling and molecular docking. (DOCX)

S3 Table Statistical variations of common feature pharmacophore models. (DOCX)

S4 Table Complete details of drug target genes corresponding to proposed phytochemicals used in network pharmacological analysis. (DOCX)

S5 Table GOLD docking scores of proposed medicinal compounds with additional phytochemicals docked in the active cavity of 3CL^pro^. (DOCX)

S6 Table GOLD docking scores of proposed medicinal compounds with additional phytochemicals docked in the active cavity of PL^pro^. (DOCX)

S1 Fig a) 3D-QSAR pharmacophore exhibits four common features consisting of 1 hydrogen bond acceptor (HBA), 2 hydrophobic (HYD), and 1 ring aromatic (RA) b) 3D-QSAR pharmacophore model with distance between chemical features. c) The most active medicinal compound; xanthoangelol_E from the training set mapped with the highest FitValue of 4.36 d) The top compound, vilazodone mapped against 3D-QSAR pharmacophore with the highest FitValue of 3.56 from the test set. e) The second top compound lapitinib with a FitValue of 3.54 mapped against 3D-QSAR pharmacophore from the test set.
(TIF)

S2 Fig a) Common feature pharmacophore results of *Senna* compounds exhibit four common features consisting of 3 hydrogen bond acceptors (HBA) and 1 ring aromatic (RA) features b) Common feature pharmacophore model with distance between chemical features. c) Isoquercetin mapped the common feature pharmacophore with highest FitValue of 0.99 from the training set. d) Compound with lowest FitValue.
(TIF)

S3 Fig Preferred binding mode of active phytochemicals in the binding site of 3CL^pro^ depicting two-dimensional (2D) docked complex with a) Xanthoangelol_E having an IC_50_ value 11.4 ±1.4 μM b) Hesperetin having an IC_50_ value 8.3 μM c) Beta-sitosterol with an IC_50_ value 1210 μM.
(TIF)

S4 Fig Preferred binding mode of active phytochemicals in the binding site of 3CL^pro^ depicting two-dimensional (2D) docked complex with a) Luteolin-7-O-glucopyranoside b) Calceolarioside_B c) Isoquercetin.
(TIF)

S5 Fig Insights into the MD simulations of vizimpro, hesperetin, beta-sitosterol, and xanthoangelol_E for the time period of 200 ns each complex. a) RMSD in complex with 3CL^pro^ b) Radius of gyration c) RMSF of 3CL^pro^ residues d) Beta-factor.
(TIF)

S6 Fig Insights into the MD simulations of vizimpro, luteolin, calceolarioside_B, and isoquercetin for the time period of 200 ns each complex a) RMSD in complex with 3CL^pro^ b) Radius of gyration c) RMSF d) Beta-factor.
(TIF)

S7 Fig RMSD graphs of extended MD simulations of compounds that exhibited instability till 200 ns. a) 100 ns of extended MD simulations of xanthoangelol_E b) 200 ns of extended MD simulations of calceolarioside_B c) 100 ns of extended MD simulations of isoquercetin.
(TIF)

S8 Fig RMSD of 50 ns MD simulations of corylifol_A and papyriflavonol_A docked at PL^pro^ active site. (TIF)
