# Supplementary material for: Senna makki and other active phytochemicals: Myths and realities behind covid19 therapeutic interventions
Source: PLoS One. 2022 Jun 14;17(6):e0268454. doi: 10.1371/journal.pone.0268454 (PMC9197063; doi:10.1371/journal.pone.0268454)
Supplement: S1 Table — (DOCX) [file pone.0268454.s001.docx]

**S1 Table.** Small molecule medicinal inhibitors of 3CL^pro^ with reported activity (IC_50_ values) against SARS-CoV-2 selected as training set for 3D-QSAR pharmacophore modeling.

| **Sr.** | **Pubchem Compound ID** | **Compound Name** | **Chemical Group** | **3CLpro IC_50_** | **Structure** |
| --- | --- | --- | --- | --- | --- |
| 1 | 10,022,050 | Xanthoangelol_E | Chalcone | 11.4 ±1.4 µM | 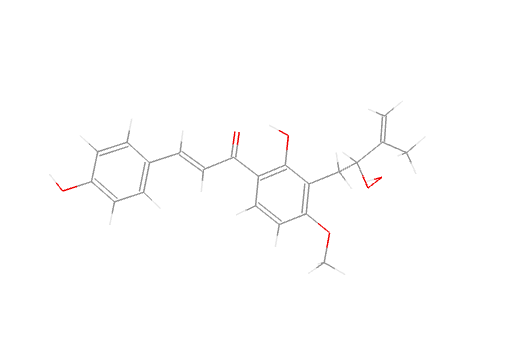 |
| 2 | 222,284 | Beta-sisterol | Phytosterol | 1210 μM | 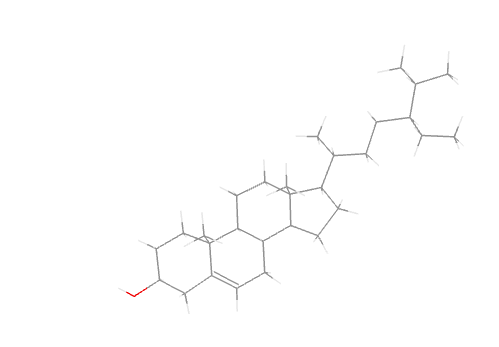 |
| 3 | 72,281 | Hesperetin | Flavonoid | 8.3 μM | 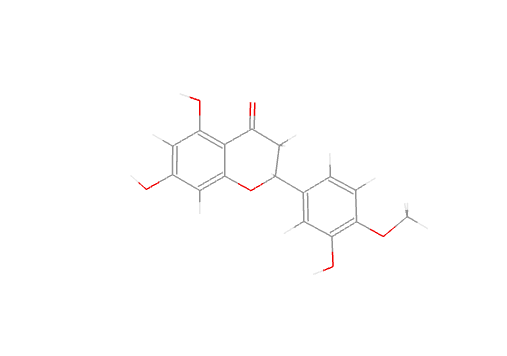 |
| 4 | 23,682,211 | Sinigrin | Polyphenol | 217 μM | 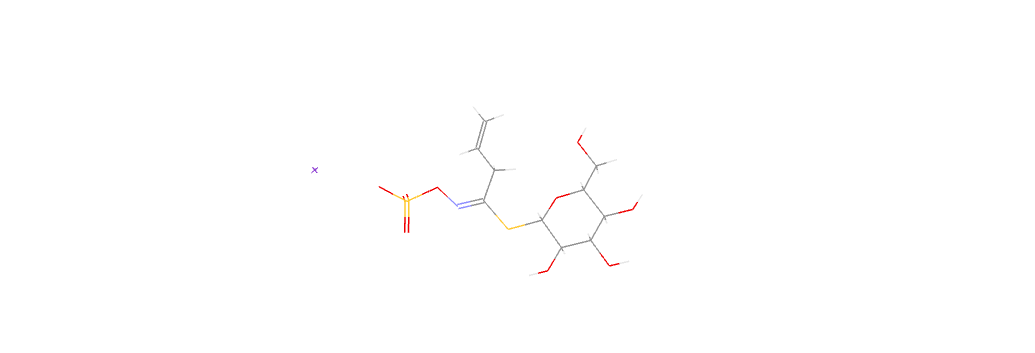 |
| 5 | 10,207 | Aloe emodin | anthraquinone | 366 μM | 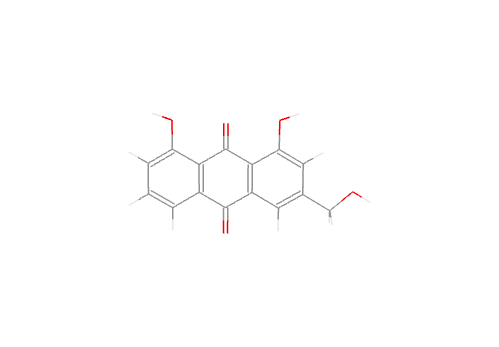 |
| 6 | 162,727 | Iguesterin | Triterpene | 22.6 ± 0.3 μM | 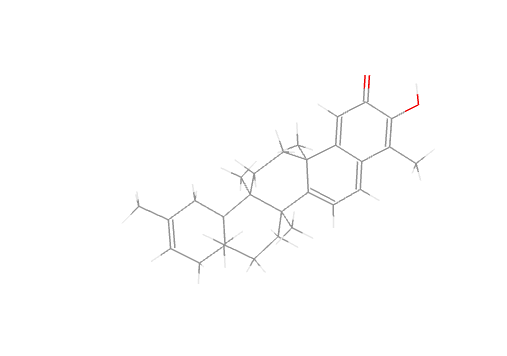 |
| 7 | 159,516 | Pristimererin | Triterpene | 5.5 ± 0.7 μM | 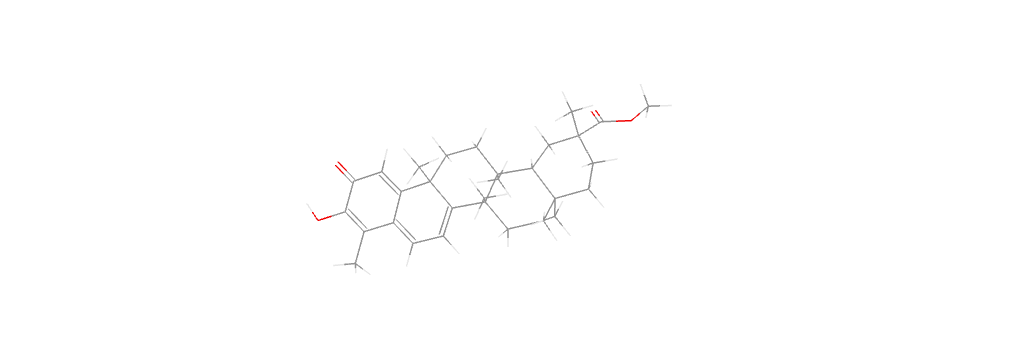 |
| 8 | 5,281,600 | Amentoflavone | Flavonoid | 8.3 μM | 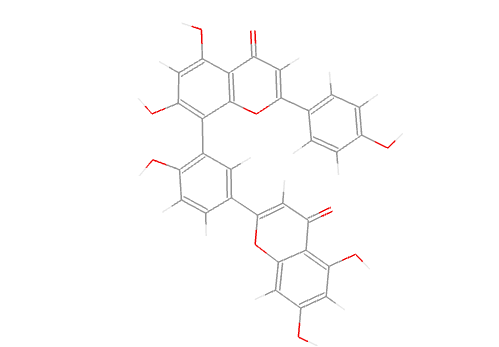 |
| 9 | 5,280,445 | Luteolin | Flavonoid | 20.2 μM | 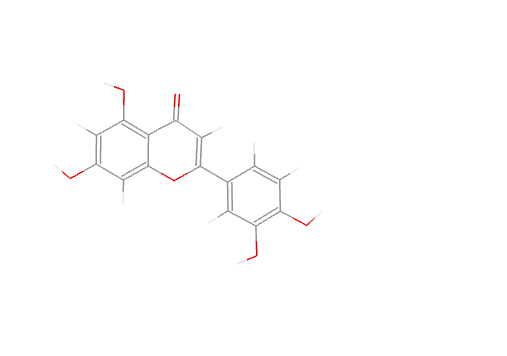 |
| 10 | 101,520 | Tingenone | Triterpene | 9.9 ± 0.1 μM | 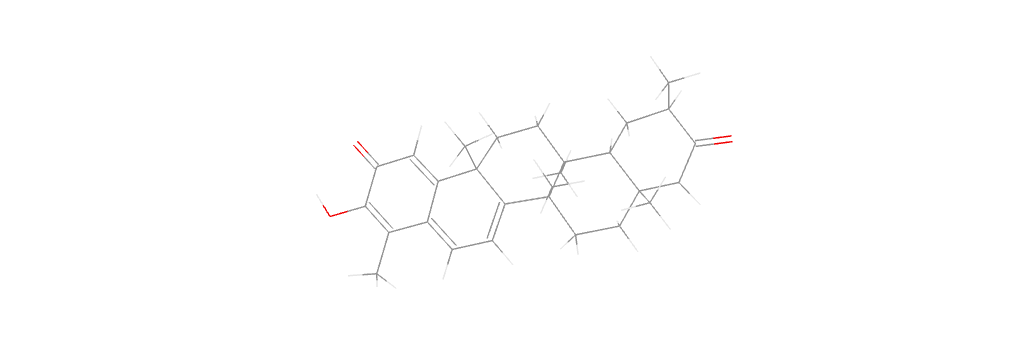 |
| 11 | 442,027 | Ferruginol | diterpenoids | 49.6 μM | 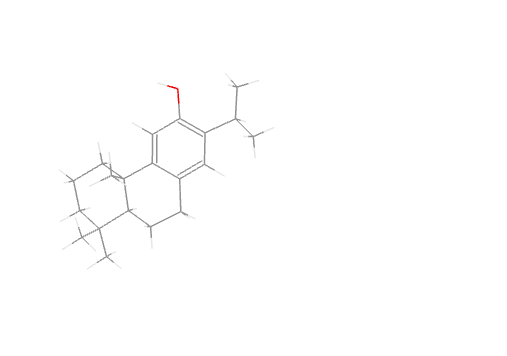 |
| 12 | 64,971 | Betulinic Acid | Triterpene | 10 μM | 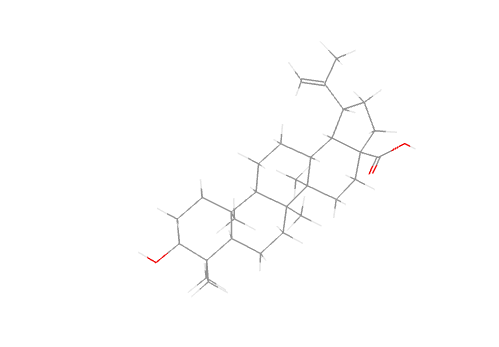 |
| 13 | 122,724 | Celastrol | Triterpene | 10.3 μM | 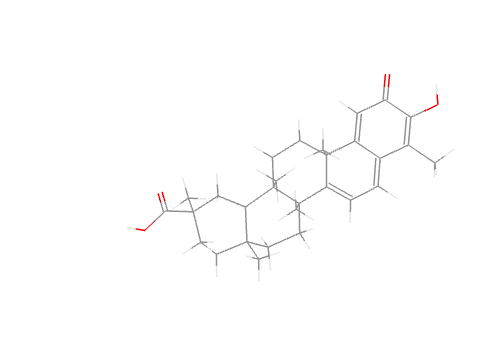 |
| 14 | 164,676 | Dihydrotanshinone I | Diterpene | 4.9 ± 1.2 μM | 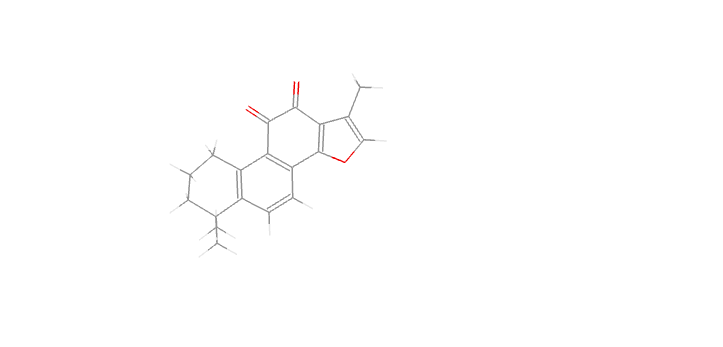 |
| 15 | 5,281,255 | Isobavachalcone | Flavonoid | 7.3 ± 0.8 μM | 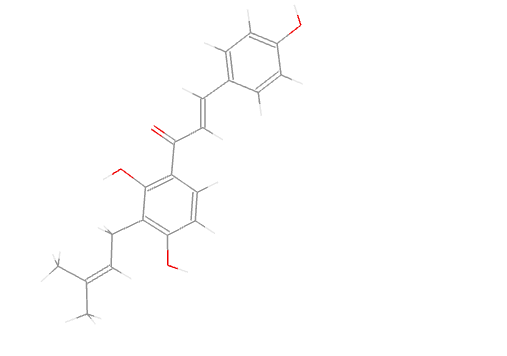 |
| 16 | 5,281,867 | Savinin | lignoid | 25 μM | 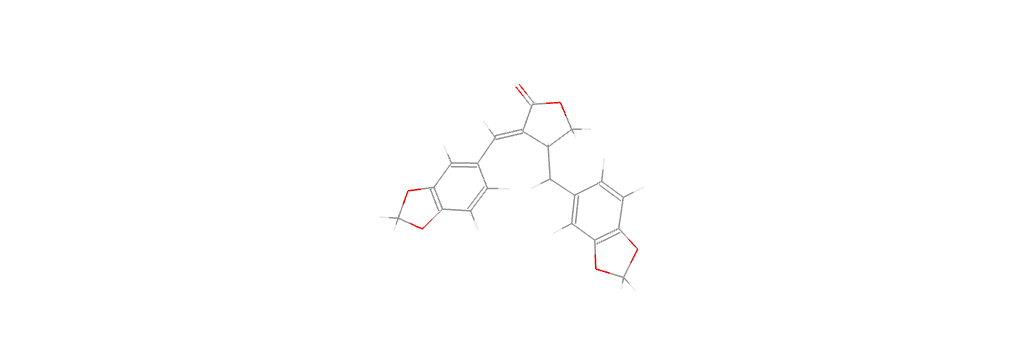 |
| 17 | 5,282,150 | Rhoifolin | Flavonoid | 27.45 μM | 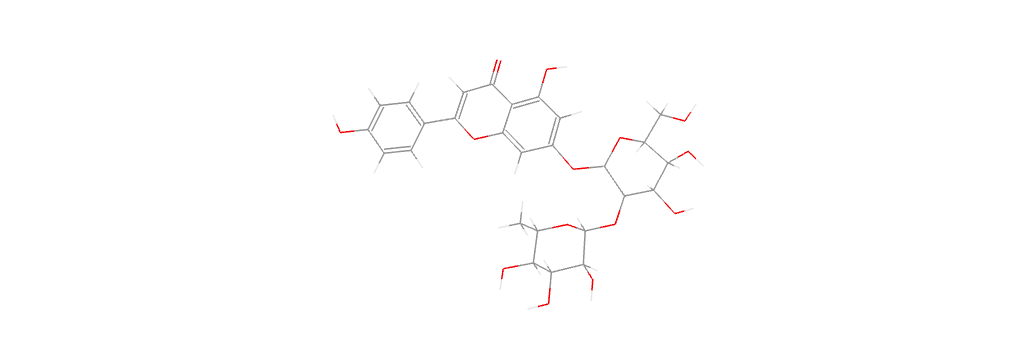 |
| 18 | 969,516 | Curcumin | Diarylheptanoid | 40 μM | 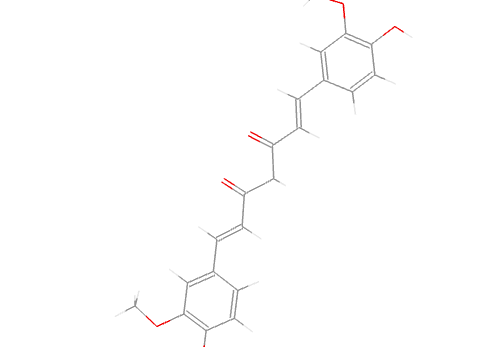 |
| 19 | 442,879 | Hinokinin | Lignans | 110 μM | 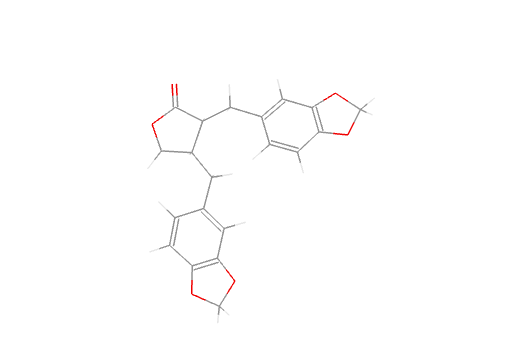 |
| 20 | 6,438,825 | Broussochalcone | Flavonoid | 57.8 ± 0.5 μM | 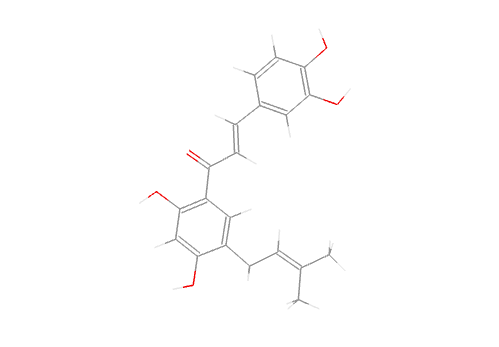 |
| 21 | 5,280,343 | Quercetin | Flavonoid | 23.8 μM | 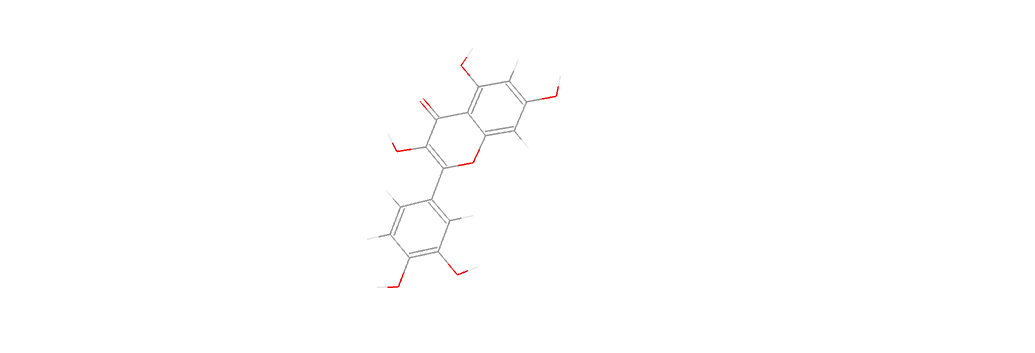 |
| 22 | 160,142 | Rosmariquinone | Diterpene | 21.1 & 30.0 µM | 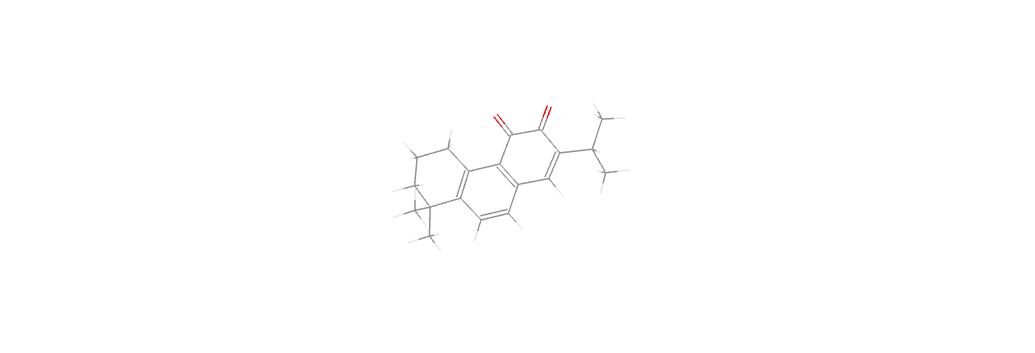 |
| 23 | 5,280,544 | Herbacetin | Flavonoid | 33.17-40.59 µM | 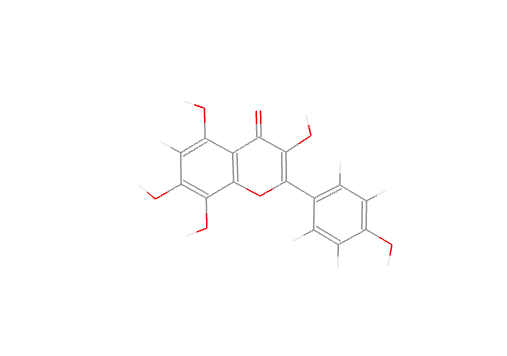 |
| 24 | 114,917 | Tanshinone I | Diterpene | 38.7 & 8.8 µM | 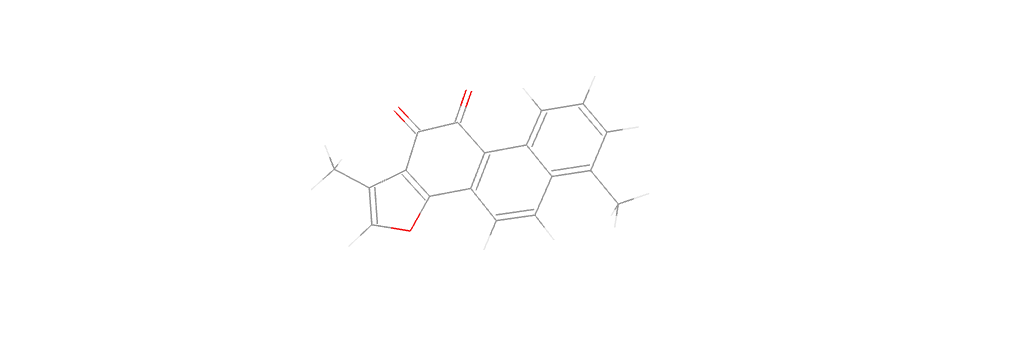 |
| 25 | 160,254 | Cryptotanshinone | Diterpene | 226.7 μM | 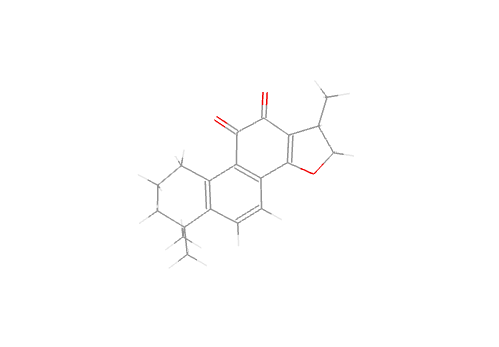 |
| 26 | 168,849 | Pectolinarin | Flavonoid | 37.78 μM | 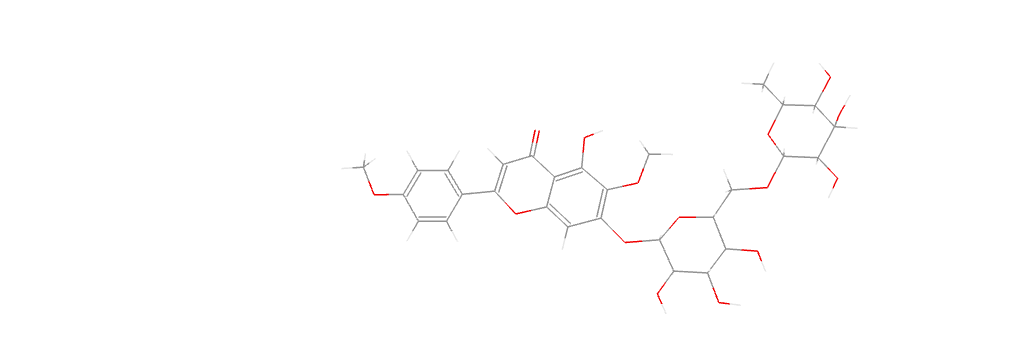 |
| 27 | 65,064 | Epigallocatechin gallate | Flavonoid | 73 μM | 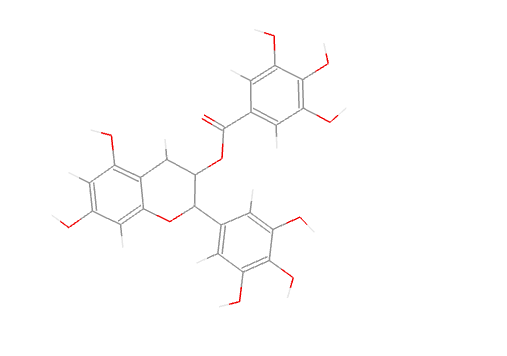 |
