# Supplementary material for: Senna makki and other active phytochemicals: Myths and realities behind covid19 therapeutic interventions
Source: PLoS One. 2022 Jun 14;17(6):e0268454. doi: 10.1371/journal.pone.0268454 (PMC9197063; doi:10.1371/journal.pone.0268454)
Supplement: S2 Table — (DOCX) [file pone.0268454.s002.docx]

**S2 Table.** Chemical compounds of *Senna alexandrina* of *Cassia senna* and *Tinnevelly senna* of *Cassia angustifolia* used for common feature pharmacophore modeling and molecular docking.

| **No.** | **Compound Name** | **_PubChem Compound ID_** | **Reference** |
| --- | --- | --- | --- |
| 1 | Caffeic acid | 689043 | [1] |
| 2 | Cynaroside | 5280637 | [2] |
| 3 | Gallic acid | 370 | [3] |
| 4 | Benzoic acid | 3469 | [4] |
| 5 | Kaempferol | 5280863 | [5] |
| 6 | Isorhamnetin | 5281654 | [6] |
| 7 | Rheinanthrone | 119396 | N/A |
| 8 | Aloe-emodin-8-O-beta-D-glucopyranoside | 5317644 | N/A |
| 9 | Salicylic acid | 338 | N/A |
| 11 | Syringic acid | 10742 | N/A |
| 12 | Sennoside A | 73111 | N/A |
| 13 | Sennoside B | 91440 | N/A |
| 14 | Sennoside C | 46173829 | N/A |
| 15 | Sennoside D | 46173830 | N/A |
| 16 | Tinnevellin glucoside | 157631 | N/A |
| 17 | 6-Hydroxymusicin glucoside | 101656110 | N/A |

Phytoconstituents having potential against different types of infections are referred in the table.

N/A signifies no data available

**References**

1. Ikeda K, Tsujimoto K, Uozaki M, Nishide M, Suzuki Y, Koyama AH, et al. Inhibition of multiplication of herpes simplex virus by caffeic acid. Int J Mol Med. 2011;28: 595–598. doi:10.3892/ijmm.2011.739

2. Tabrez S, Rahman F, Ali R, Alouffi AS, Akand SK, Alshehri BM, et al. Cynaroside inhibits Leishmania donovani UDP-galactopyranose mutase and induces reactive oxygen species to exert antileishmanial response. Biosci Rep. 2021;41. doi:10.1042/BSR20203857

3. Choi HJ, Song JH, Bhatt LR, Baek SH. Anti-human rhinovirus activity of gallic acid possessing antioxidant capacity. Phytother Res. 2010;24: 1292–1296. doi:10.1002/ptr.3101

4. Strand M, Islam K, Edlund K, Oberg CT, Allard A, Bergström T, et al. 2-[4,5-Difluoro-2-(2-fluorobenzoylamino)-benzoylamino]benzoic acid, an antiviral compound with activity against acyclovir-resistant isolates of herpes simplex virus types 1 and 2. Antimicrob Agents Chemother. 2012;56: 5735–5743. doi:10.1128/AAC.01072-12

5. Ürményi FGG, Saraiva G do N, Casanova LM, Matos ADS, de Magalhães Camargo LM, Romanos MTV, et al. Anti-HSV-1 and HSV-2 Flavonoids and a New Kaempferol Triglycoside from the Medicinal Plant Kalanchoe daigremontiana. Chem Biodivers. 2016;13: 1707–1714. doi:10.1002/cbdv.201600127

6. Abdal Dayem A, Choi HY, Kim YB, Cho S-G. Antiviral effect of methylated flavonol isorhamnetin against influenza. PLoS One. 2015;10: e0121610. doi:10.1371/journal.pone.0121610
