# Supplementary material for: Senna makki and other active phytochemicals: Myths and realities behind covid19 therapeutic interventions
Source: PLoS One. 2022 Jun 14;17(6):e0268454. doi: 10.1371/journal.pone.0268454 (PMC9197063; doi:10.1371/journal.pone.0268454)
Supplement: S3 Table — (DOCX) [file pone.0268454.s003.docx]

**S3 Table.** Statistical variations of common feature pharmacophore models.

| **Validation with known Active/Inactive compounds** | | | | | | | | |
| --- | --- | --- | --- | --- | --- | --- | --- | --- |
| **Pharmacophore** | **Total Active** | **Total Inactive** | **True Positives** | **True Negatives** | **False Positives** | **False Negatives** | **Sensitivity** | **Specificity** |
| 1 | 77 | 39 | 30 | 14 | 25 | 47 | 0.38 | 0.35 |
| 2 | 77 | 39 | 31 | 14 | 25 | 46 | 0.40 | 0.35 |
| 3 | 77 | 39 | 33 | 14 | 25 | 44 | 0.42 | 0.35 |
| 4 | 77 | 39 | 32 | 14 | 25 | 45 | 0.41 | 0.35 |
| 5 | 77 | 39 | 33 | 11 | 28 | 44 | 0.42 | 0.28 |
| 6 | 77 | 39 | 33 | 11 | 28 | 44 | 0.42 | 0.28 |
| 7 | 77 | 39 | 34 | 10 | 29 | 43 | 0.44 | 0.25 |
| 8 | 77 | 39 | 33 | 14 | 25 | 44 | 0.42 | 0.35 |
| 9 | 77 | 39 | 31 | 15 | 24 | 46 | 0.40 | 0.38 |
| 10 | 77 | 39 | 34 | 14 | 25 | 43 | 0.44 | 0.35 |
