# Supplementary material for: Senna makki and other active phytochemicals: Myths and realities behind covid19 therapeutic interventions
Source: PLoS One. 2022 Jun 14;17(6):e0268454. doi: 10.1371/journal.pone.0268454 (PMC9197063; doi:10.1371/journal.pone.0268454)
Supplement: S4 Table — (DOCX) [file pone.0268454.s004.docx]

**S4 Table.** Complete details of drug target genes corresponding to proposed phytochemicals used in Network pharmacological analysis.

| **Xanthoangelol_E** | | | | |
| --- | --- | --- | --- | --- |
| **Target** | **Common name** | **Uniprot ID** | **ChEMBL ID** | **Target Class** |
| Arachidonate 5-lipoxygenase | ALOX5 | P09917 | CHEMBL215 | Oxidoreductase |
| 3-phosphoinositide dependent protein kinase-1 | PDPK1 | O15530 | CHEMBL2534 | Kinase |
| Thrombin and coagulation factor X | F10 | P00742 | CHEMBL244 | Protease |
| Thrombin | F2 | P00734 | CHEMBL204 | Protease |
| Protein kinase C beta | PRKCB | P05771 | CHEMBL3045 | Kinase |
| Protein kinase C eta | PRKCH | P24723 | CHEMBL3616 | Kinase |
| Serine/threonine-protein kinase AKT | AKT1 | P31749 | CHEMBL4282 | Kinase |
| Cyclooxygenase-1 | PTGS1 | P23219 | CHEMBL221 | Oxidoreductase |
|  |  |  |  |  |
| **Beta-sitosterol** | | | | |
| **Target** | **Common name** | **Uniprot ID** | **ChEMBL ID** | **Target Class** |
| HMG-CoA reductase | HMGCR | P04035 | CHEMBL402 | Oxidoreductase |
| Cytochrome P450 51 (by homology) | CYP51A1 | Q16850 | CHEMBL3849 | Cytochrome P450 |
| Androgen Receptor | AR | P10275 | CHEMBL1871 | Nuclear receptor |
| Niemann-Pick C1-like protein 1 | NPC1L1 | Q9UHC9 | CHEMBL2027 | Other membrane protein |
| LXR-alpha | NR1H3 | Q13133 | CHEMBL2808 | Nuclear receptor |
| Cytochrome P450 17A1 | CYP17A1 | P05093 | CHEMBL3522 | Cytochrome P450 |
| Nuclear receptor ROR-gamma | RORC | P51449 | CHEMBL1741186 | Nuclear receptor |
| Cytochrome P450 19A1 | CYP19A1 | P11511 | CHEMBL1978 | Cytochrome P450 |
| Estrogen receptor beta | ESR2 | Q92731 | CHEMBL242 | Nuclear receptor |
| Estrogen receptor alpha | ESR1 | P03372 | CHEMBL206 | Nuclear receptor |
| Testis-specific androgen-binding protein | SHBG | P04278 | CHEMBL3305 | Secreted protein |
| Sterol regulatory element-binding protein 2 | SREBF2 | Q12772 | CHEMBL1795166 | Unclassified protein |
| Cytochrome P450 2C19 | CYP2C19 | P33261 | CHEMBL3622 | Cytochrome P450 |
| Norepinephrine transporter | SLC6A2 | P23975 | CHEMBL222 | Electrochemical transporter |
| Butyrylcholinesterase | BCHE | P06276 | CHEMBL1914 | Hydrolase |
| Nuclear receptor ROR-alpha | RORA | P35398 | CHEMBL5868 | Nuclear receptor |
| Protein-tyrosine phosphatase 1B | PTPN1 | P18031 | CHEMBL335 | Phosphatase |
| Corticosteroid binding globulin | SERPINA6 | P08185 | CHEMBL2421 | Secreted protein |
| Serotonin transporter | SLC6A4 | P31645 | CHEMBL228 | Electrochemical transporter |
| Glucose-6-phosphate 1-dehydrogenase | G6PD | P11413 | CHEMBL5347 | Enzyme |
| Nuclear receptor subfamily 1 group I member 3 (by homology) | NR1I3 | Q14994 | CHEMBL5503 | Nuclear receptor |
| Muscarinic acetylcholine receptor M2 | CHRM2 | P08172 | CHEMBL211 | Family A G protein-coupled receptor |
| Acetylcholinesterase | ACHE | P22303 | CHEMBL220 | Hydrolase |
| Vitamin D receptor | VDR | P11473 | CHEMBL1977 | Nuclear receptor |
| Carboxylesterase 2 | CES2 | O00748 | CHEMBL3180 | Enzyme |
| LXR-beta | NR1H2 | P55055 | CHEMBL4093 | Nuclear receptor |
| Dual specificity phosphatase Cdc25A | CDC25A | P30304 | CHEMBL3775 | Phosphatase |
| Prostanoid EP1 receptor (by homology) | PTGER1 | P34995 | CHEMBL1811 | Family A G protein-coupled receptor |
| Prostanoid EP2 receptor (by homology) | PTGER2 | P43116 | CHEMBL1881 | Family A G protein-coupled receptor |
|  |  |  |  |  |
| **Hesperetin** | | | | |
| **Target** | **Common name** | **Uniprot ID** | **ChEMBL ID** | **Target Class** |
| Carbonic anhydrase VII | CA7 | P43166 | CHEMBL2326 | Lyase |
| Carbonic anhydrase XII | CA12 | O43570 | CHEMBL3242 | Lyase |
| Carbonic anhydrase IV | CA4 | P22748 | CHEMBL3729 | Lyase |
| Cytochrome P450 1B1 | CYP1B1 | Q16678 | CHEMBL4878 | Cytochrome P450 |
| Cytochrome P450 19A1 | CYP19A1 | P11511 | CHEMBL1978 | Cytochrome P450 |
| Taste receptor type 2 member 31 | TAS2R31 | P59538 | CHEMBL2034804 | Taste family G protein-coupled receptor |
| Adenosine A1 receptor (by homology) | ADORA1 | P30542 | CHEMBL226 | Family A G protein-coupled receptor |
| Adenosine A3 receptor | ADORA3 | P0DMS8 | CHEMBL256 | Family A G protein-coupled receptor |
| ATP-binding cassette sub-family G member 2 | ABCG2 | Q9UNQ0 | CHEMBL5393 | Primary active transporter |
| Estradiol 17-beta-dehydrogenase 1 | HSD17B1 | P14061 | CHEMBL3181 | Enzyme |
| Estrogen receptor beta | ESR2 | Q92731 | CHEMBL242 | Nuclear receptor |
| Estrogen receptor alpha | ESR1 | P03372 | CHEMBL206 | Nuclear receptor |
| Monoamine oxidase B | MAOB | P27338 | CHEMBL2039 | Oxidoreductase |
| Multidrug resistance-associated protein 1 | ABCC1 | P33527 | CHEMBL3004 | Primary active transporter |
| Testis-specific androgen-binding protein | SHBG | P04278 | CHEMBL3305 | Secreted protein |
| Carbonyl reductase [NADPH] 1 | CBR1 | P16152 | CHEMBL5586 | Enzyme |
| Matrix metalloproteinase 13 | MMP13 | P45452 | CHEMBL280 | Protease |
| Cyclooxygenase-1 | PTGS1 | P23219 | CHEMBL221 | Oxidoreductase |
| Beta-secretase 1 | BACE1 | P56817 | CHEMBL4822 | Protease |
| Matrix metalloproteinase 12 | MMP12 | P39900 | CHEMBL4393 | Protease |
| Metabotropic glutamate receptor 5 | GRM5 | P41594 | CHEMBL3227 | Family C G protein-coupled receptor |
| Tyrosine-protein kinase SRC | SRC | P12931 | CHEMBL267 | Kinase |
| Aldo-keto-reductase family 1 member C3 | AKR1C3 | P42330 | CHEMBL4681 | Enzyme |
| Phospholipase A2 group 1B | PLA2G1B | P04054 | CHEMBL4426 | Enzyme |
| Kallikrein 1 | KLK1 | P06870 | CHEMBL2319 | Protease |
| Kallikrein 2 | KLK2 | P20151 | CHEMBL2442 | Protease |
| Carbonic anhydrase III | CA3 | P07451 | CHEMBL2885 | Lyase |
| Neuronal acetylcholine receptor protein alpha-7 subunit | CHRNA7 | P36544 | CHEMBL2492 | Ligand-gated ion channel |
| Telomerase reverse transcriptase | TERT | O14746 | CHEMBL2916 | Enzyme |
| Carbonic anhydrase II | CA2 | P00918 | CHEMBL205 | Lyase |
| Carbonic anhydrase I | CA1 | P00915 | CHEMBL261 | Lyase |
| Carbonic anhydrase VI | CA6 | P23280 | CHEMBL3025 | Lyase |
| Carbonic anhydrase VA | CA5A | P35218 | CHEMBL4789 | Lyase |
| DNA polymerase beta (by homology) | POLB | P06746 | CHEMBL2392 | Enzyme |
| Plasminogen activator inhibitor-1 | SERPINE1 | P05121 | CHEMBL3475 | Secreted protein |
| Beta amyloid A4 protein | APP | P05067 | CHEMBL2487 | Membrane receptor |
| Phospholipase A2 group V | PLA2G5 | P39877 | CHEMBL4323 | Enzyme |
| Group X secretory phospholipase A2 | PLA2G10 | O15496 | CHEMBL4342 | Enzyme |
| Plasminogen | PLG | P00747 | CHEMBL1801 | Protease |
| NADPH oxidase 4 | NOX4 | Q9NPH5 | CHEMBL1250375 | Enzyme |
| Carbonic anhydrase XIII | CA13 | Q8N1Q1 | CHEMBL3912 | Lyase |
| Carbonic anhydrase VB | CA5B | Q9Y2D0 | CHEMBL3969 | Lyase |
| Acetylcholinesterase | ACHE | P22303 | CHEMBL220 | Hydrolase |
| Beta-glucuronidase | GUSB | P08236 | CHEMBL2728 | Enzyme |
| Acyl coenzyme A:cholesterol acyltransferase | CES1 | P23141 | CHEMBL2265 | Enzyme |
| Sodium/glucose cotransporter 2 | SLC5A2 | P31639 | CHEMBL3884 | Electrochemical transporter |
| Carboxylesterase 2 | CES2 | O00748 | CHEMBL3180 | Enzyme |
| Retinoid X receptor alpha | RXRA | P19793 | CHEMBL2061 | Nuclear receptor |
| Vascular endothelial growth factor receptor 2 | KDR | P35968 | CHEMBL279 | Kinase |
| Metabotropic glutamate receptor 2 (by homology) | GRM2 | Q14416 | CHEMBL5137 | Family C G protein-coupled receptor |
| Aldose reductase | AKR1B1 | P15121 | CHEMBL1900 | Enzyme |
| Cyclin-dependent kinase 1/cyclin B1 | CDK1 CCNB1 | P06493 P14635 | CHEMBL1907602 | Other cytosolic protein |
| Cyclin-dependent kinase 2/cyclin E1 | CCNE1 CDK2 | P24864 P24941 | CHEMBL1907605 | Kinase |
| Dual specificity protein phosphatase 3 | DUSP3 | P51452 | CHEMBL2635 | Phosphatase |
| Nerve growth factor receptor Trk-A | NTRK1 | P04629 | CHEMBL2815 | Kinase |
| Serine/threonine-protein kinase Aurora-A | AURKA | O14965 | CHEMBL4722 | Kinase |
| Carbonic anhydrase IX | CA9 | Q16790 | CHEMBL3594 | Lyase |
| DNA topoisomerase I | TOP1 | P11387 | CHEMBL1781 | Isomerase |
| DNA (cytosine-5)-methyltransferase 1 | DNMT1 | P26358 | CHEMBL1993 | Writer |
| 6-phosphogluconate dehydrogenase | PGD | P52209 | CHEMBL3404 | Enzyme |
|  |  |  |  |  |
| **Calceolarioside-B** | | | | |
| **Target** | **Common name** | **Uniprot ID** | **ChEMBL ID** | **Target Class** |
| Protein kinase C alpha | PRKCA | P17252 | CHEMBL299 | Kinase |
| Matrix metalloproteinase 12 | MMP12 | P39900 | CHEMBL4393 | Protease |
| Matrix metalloproteinase 2 | MMP2 | P08253 | CHEMBL333 | Protease |
| Aldose reductase | AKR1B1 | P15121 | CHEMBL1900 | Enzyme |
| Heat shock protein HSP 90-alpha | HSP90AA1 | P07900 | CHEMBL3880 | Other cytosolic protein |
| Adenosine A2a receptor (by homology) | ADORA2A | P29274 | CHEMBL251 | Family A G protein-coupled receptor |
| Matrix metalloproteinase 13 | MMP13 | P45452 | CHEMBL280 | Protease |
| Aldehyde dehydrogenase | ALDH2 | P05091 | CHEMBL1935 | Oxidoreductase |
| Adenosine A3 receptor | ADORA3 | P0DMS8 | CHEMBL256 | Family A G protein-coupled receptor |
| TNF-alpha | TNF | P01375 | CHEMBL1825 | Secreted protein |
| Coagulation factor VII/tissue factor | F3 | P13726 | CHEMBL4081 | Surface antigen |
| Tyrosyl-tRNA synthetase | YARS | P54577 | CHEMBL3179 | Enzyme |
| Interleukin-1 receptor-associated kinase 4 | IRAK4 | Q9NWZ3 | CHEMBL3778 | Kinase |
| Solute carrier family 28 member 3 | SLC28A3 | Q9HAS3 | CHEMBL5707 | Electrochemical transporter |
| Sodium/nucleoside cotransporter 2 | SLC28A2 | O43868 | CHEMBL5780 | Electrochemical transporter |
| Beta amyloid A4 protein | APP | P05067 | CHEMBL2487 | Membrane receptor |
| Aldo-keto reductase family 1 member B10 | AKR1B10 | O60218 | CHEMBL5983 | Enzyme |
| Matrix metalloproteinase 7 | MMP7 | P09237 | CHEMBL4073 | Protease |
| Matrix metalloproteinase 8 | MMP8 | P22894 | CHEMBL4588 | Protease |
| Equilibrative nucleoside transporter 1 | SLC29A1 | Q99808 | CHEMBL1997 | Electrochemical transporter |
| 6-O-methylguanine-DNA methyltransferase | MGMT | P16455 | CHEMBL2864 | Enzyme |
| Matrix metalloproteinase 1 | MMP1 | P03956 | CHEMBL332 | Protease |
| Inosine-5'-monophosphate dehydrogenase 1 | IMPDH1 | P20839 | CHEMBL1822 | Oxidoreductase |
| cAMP-dependent protein kinase alpha-catalytic subunit | PRKACA | P17612 | CHEMBL4101 | Kinase |
|  |  |  |  |  |
| **Luteolin** | | | | |
| **Target** | **Common name** | **Uniprot ID** | **ChEMBL ID** | **Target Class** |
| NADPH oxidase 4 | NOX4 | Q9NPH5 | CHEMBL1250375 | Enzyme |
| Aldose reductase | AKR1B1 | P15121 | CHEMBL1900 | Enzyme |
| Cyclin-dependent kinase 5/CDK5 activator 1 | CDK5R1 CDK5 | Q15078 Q00535 | CHEMBL1907600 | Kinase |
| Xanthine dehydrogenase | XDH | P47989 | CHEMBL1929 | Oxidoreductase |
| Monoamine oxidase A | MAOA | P21397 | CHEMBL1951 | Oxidoreductase |
| Tyrosine-protein kinase receptor FLT3 | FLT3 | P36888 | CHEMBL1974 | Kinase |
| Carbonic anhydrase II | CA2 | P00918 | CHEMBL205 | Lyase |
| Cyclin-dependent kinase 1/cyclin B | CCNB3 CDK1 CCNB1 CCNB2 | Q8WWL7 P06493 P14635 O95067 | CHEMBL2094127 | Other cytosolic protein |
| Arachidonate 5-lipoxygenase | ALOX5 | P09917 | CHEMBL215 | Oxidoreductase |
| Adenosine A1 receptor (by homology) | ADORA1 | P30542 | CHEMBL226 | Family A G protein-coupled receptor |
| Carbonic anhydrase VII | CA7 | P43166 | CHEMBL2326 | Lyase |
| Glyoxalase I | GLO1 | Q04760 | CHEMBL2424 | Enzyme |
| Beta amyloid A4 protein | APP | P05067 | CHEMBL2487 | Membrane receptor |
| Tyrosine-protein kinase SYK | SYK | P43405 | CHEMBL2599 | Kinase |
| Glycogen synthase kinase-3 beta | GSK3B | P49841 | CHEMBL262 | Kinase |
| Poly [ADP-ribose] polymerase-1 | PARP1 | P09874 | CHEMBL3105 | Enzyme |
| Transthyretin | TTR | P02766 | CHEMBL3194 | Secreted protein |
| Matrix metalloproteinase 9 | MMP9 | P14780 | CHEMBL321 | Protease |
| Carbonic anhydrase XII | CA12 | O43570 | CHEMBL3242 | Lyase |
| Matrix metalloproteinase 2 | MMP2 | P08253 | CHEMBL333 | Protease |
| Carbonic anhydrase IV | CA4 | P22748 | CHEMBL3729 | Lyase |
| Matrix metalloproteinase 12 | MMP12 | P39900 | CHEMBL4393 | Protease |
| Lymphocyte differentiation antigen CD38 | CD38 | P28907 | CHEMBL4660 | Enzyme |
| Cytochrome P450 1B1 | CYP1B1 | Q16678 | CHEMBL4878 | Cytochrome P450 |
| ATP-binding cassette sub-family G member 2 | ABCG2 | Q9UNQ0 | CHEMBL5393 | Primary active transporter |
| Aldo-keto reductase family 1 member B10 | AKR1B10 | O60218 | CHEMBL5983 | Enzyme |
| Tankyrase-2 | TNKS2 | Q9H2K2 | CHEMBL6154 | Enzyme |
| Tankyrase-1 | TNKS | O95271 | CHEMBL6164 | Enzyme |
| DNA topoisomerase I (by homology) | TOP1 | P11387 | CHEMBL1781 | Isomerase |
| Arginase-1 (by homology) | ARG1 | P05089 | CHEMBL1075097 | Enzyme |
| Receptor-type tyrosine-protein phosphatase S | PTPRS | Q13332 | CHEMBL2396508 | Phosphatase |
| Multidrug resistance-associated protein 1 | ABCC1 | P33527 | CHEMBL3004 | Primary active transporter |
| Estradiol 17-beta-dehydrogenase 1 | HSD17B1 | P14061 | CHEMBL3181 | Enzyme |
| Acetylcholinesterase | ACHE | P22303 | CHEMBL220 | Hydrolase |
| Cyclin-dependent kinase 6 | CDK6 | Q00534 | CHEMBL2508 | Kinase |
| P-glycoprotein 1 | ABCB1 | P08183 | CHEMBL4302 | Primary active transporter |
| Estradiol 17-beta-dehydrogenase 2 | HSD17B2 | P37059 | CHEMBL2789 | Enzyme |
| Cytochrome P450 19A1 | CYP19A1 | P11511 | CHEMBL1978 | Cytochrome P450 |
| Estrogen receptor beta | ESR2 | Q92731 | CHEMBL242 | Nuclear receptor |
| Adenosine A2a receptor (by homology) | ADORA2A | P29274 | CHEMBL251 | Family A G protein-coupled receptor |
| Casein kinase II alpha | CSNK2A1 | P68400 | CHEMBL3629 | Kinase |
| Arachidonate 15-lipoxygenase | ALOX15 | P16050 | CHEMBL2903 | Enzyme |
| Arachidonate 12-lipoxygenase | ALOX12 | P18054 | CHEMBL3687 | Enzyme |
| Estrogen receptor alpha | ESR1 | P03372 | CHEMBL206 | Nuclear receptor |
| Cyclooxygenase-2 | PTGS2 | P35354 | CHEMBL230 | Oxidoreductase |
| Cystic fibrosis transmembrane conductance regulator | CFTR | P13569 | CHEMBL4051 | Other ion channel |
| AMY1C | AMY1A | P04745 | CHEMBL2478 | Enzyme |
| G protein-coupled receptor kinase 6 | GRK6 | P43250 | CHEMBL6144 | Kinase |
| Carbonic anhydrase I | CA1 | P00915 | CHEMBL261 | Lyase |
| Carbonic anhydrase IX | CA9 | Q16790 | CHEMBL3594 | Lyase |
| Cyclin-dependent kinase 2 | CDK2 | P24941 | CHEMBL301 | Kinase |
| Telomerase reverse transcriptase | TERT | O14746 | CHEMBL2916 | Enzyme |
| Cyclin-dependent kinase 1 | CDK1 | P06493 | CHEMBL308 | Kinase |
| Tyrosinase | TYR | P14679 | CHEMBL1973 | Oxidoreductase |
| Aryl hydrocarbon receptor | AHR | P35869 | CHEMBL3201 | Transcription factor |
| Estrogen-related receptor alpha | ESRRA | P11474 | CHEMBL3429 | Nuclear receptor |
| G-protein coupled receptor 35 | GPR35 | Q9HC97 | CHEMBL1293267 | Family A G protein-coupled receptor |
| Vasopressin V2 receptor | AVPR2 | P30518 | CHEMBL1790 | Family A G protein-coupled receptor |
| Insulin-like growth factor I receptor | IGF1R | P08069 | CHEMBL1957 | Kinase |
| Epidermal growth factor receptor erbB1 | EGFR | P00533 | CHEMBL203 | Kinase |
| Thrombin | F2 | P00734 | CHEMBL204 | Protease |
| Serine/threonine-protein kinase PIM1 | PIM1 | P11309 | CHEMBL2147 | Kinase |
| Serine/threonine-protein kinase Aurora-B | AURKB | Q96GD4 | CHEMBL2185 | Kinase |
| Dopamine D4 receptor | DRD4 | P21917 | CHEMBL219 | Family A G protein-coupled receptor |
| Myeloperoxidase | MPO | P05164 | CHEMBL2439 | Enzyme |
| PI3-kinase p85-alpha subunit | PIK3R1 | P27986 | CHEMBL2506 | Enzyme |
| Death-associated protein kinase 1 | DAPK1 | P53355 | CHEMBL2558 | Kinase |
| Liver glycogen phosphorylase | PYGL | P06737 | CHEMBL2568 | Enzyme |
| Tyrosine-protein kinase SRC | SRC | P12931 | CHEMBL267 | Kinase |
| Focal adhesion kinase 1 | PTK2 | Q05397 | CHEMBL2695 | Kinase |
| Vascular endothelial growth factor receptor 2 | KDR | P35968 | CHEMBL279 | Kinase |
| Matrix metalloproteinase 13 | MMP13 | P45452 | CHEMBL280 | Protease |
| Matrix metalloproteinase 3 | MMP3 | P08254 | CHEMBL283 | Protease |
| Carbonic anhydrase III | CA3 | P07451 | CHEMBL2885 | Lyase |
| Serine/threonine-protein kinase PLK1 | PLK1 | P53350 | CHEMBL3024 | Kinase |
| Carbonic anhydrase VI | CA6 | P23280 | CHEMBL3025 | Lyase |
| Protein kinase N1 | PKN1 | Q16512 | CHEMBL3384 | Kinase |
| Carbonic anhydrase XIV | CA14 | Q9ULX7 | CHEMBL3510 | Lyase |
| Hepatocyte growth factor receptor | MET | P08581 | CHEMBL3717 | Kinase |
| Serine/threonine-protein kinase NEK2 | NEK2 | P51955 | CHEMBL3835 | Kinase |
| Interleukin-8 receptor A | CXCR1 | P25024 | CHEMBL4029 | Family A G protein-coupled receptor |
| CaM kinase II beta | CAMK2B | Q13554 | CHEMBL4121 | Kinase |
| ALK tyrosine kinase receptor | ALK | Q9UM73 | CHEMBL4247 | Kinase |
| Serine/threonine-protein kinase AKT | AKT1 | P31749 | CHEMBL4282 | Kinase |
| Serine/threonine-protein kinase NEK6 | NEK6 | Q9HC98 | CHEMBL4309 | Kinase |
| Phospholipase A2 group 1B | PLA2G1B | P04054 | CHEMBL4426 | Enzyme |
| Carbonic anhydrase VA | CA5A | P35218 | CHEMBL4789 | Lyase |
| Beta-secretase 1 | BACE1 | P56817 | CHEMBL4822 | Protease |
| Tyrosine-protein kinase receptor UFO | AXL | P30530 | CHEMBL4895 | Kinase |
| NUAK family SNF1-like kinase 1 | NUAK1 | O60285 | CHEMBL5784 | Kinase |
| Aldo-keto reductase family 1 member C2 (by homology) | AKR1C2 | P52895 | CHEMBL5847 | Enzyme |
| Aldo-keto reductase family 1 member C1 (by homology) | AKR1C1 | Q04828 | CHEMBL5905 | Enzyme |
| Aldo-keto-reductase family 1 member C3 (by homology) | AKR1C3 | P42330 | CHEMBL4681 | Enzyme |
| Aldo-keto reductase family 1 member C4 (by homology) | AKR1C4 | P17516 | CHEMBL4999 | Enzyme |
| Carbonic anhydrase XIII (by homology) | CA13 | Q8N1Q1 | CHEMBL3912 | Lyase |
| Aldehyde reductase (by homology) | AKR1A1 | P14550 | CHEMBL2246 | Enzyme |
| 6-phosphofructo-2-kinase/fructose-2,6-bisphosphatase 3 | PFKFB3 | Q16875 | CHEMBL2331053 | Enzyme |
| Plasminogen | PLG | P00747 | CHEMBL1801 | Protease |
| Lysine-specific demethylase 4D-like | KDM4E | B2RXH2 | CHEMBL1293226 | Eraser |
| Androgen Receptor | AR | P10275 | CHEMBL1871 | Nuclear receptor |
|  |  |  |  |  |
| **Sennosides** | | | | |
| **Target** | **Common name** | **Uniprot ID** | **ChEMBL ID** | **Target Class** |
| Troponin, cardiac muscle | TNNC1 TNNT2 TNNI3 | P63316 P45379 P19429 | CHEMBL2095202 | Unclassified protein |
| Estrogen receptor alpha | ESR1 | P03372 | CHEMBL206 | Nuclear receptor |
