# Supplementary material for: Senna makki and other active phytochemicals: Myths and realities behind covid19 therapeutic interventions
Source: PLoS One. 2022 Jun 14;17(6):e0268454. doi: 10.1371/journal.pone.0268454 (PMC9197063; doi:10.1371/journal.pone.0268454)
Supplement: S5 Table — (DOCX) [file pone.0268454.s005.docx]

**S5 Table.** GOLD docking scores of proposed medicinal compounds with additional phytochemicals docked in the active cavity of 3CL^pro^.

| **Fitness** | **S(hb_ext)** | **S(vdw_ext)** | **Ligand Name** |
| --- | --- | --- | --- |
| 37.48 | 0.11 | 27.79 | Xanthoangelol_E |
| 36.07 | 0.00 | 31.36 | Hesperetin |
| 25.18 | 0.08 | 19.39 | Beta-sitoterol |
| 23.65 | 0.00 | 23.60 | Calceolarioside_B |
| -68.88 | 0.25 - | -40.39 | Isobavachalcone |
| -203.58 - | 0.03 | 138.79 | PapyriflavonolA |
