# Supplementary material for: Senna makki and other active phytochemicals: Myths and realities behind covid19 therapeutic interventions
Source: PLoS One. 2022 Jun 14;17(6):e0268454. doi: 10.1371/journal.pone.0268454 (PMC9197063; doi:10.1371/journal.pone.0268454)
Supplement: S6 Table — (DOCX) [file pone.0268454.s006.docx]

**S6 Table.** GOLD docking scores of proposed medicinal compounds with additional phytochemicals docked in the active cavity of PL^pro^.

| **Fitness** | **S(hb_ext)** | **S(vdw_ext)** | **Ligand Name** |
| --- | --- | --- | --- |
| 57.95 | 0.00 | 44.47 | Papyriflavonol_A |
| 57.70 | 0.00 | 43.60 | Corylifol_A |
| 52.91 | 0.00 | 42.10 | 4-omethylbavachalcone |
| 51.75 | 0.00 | 40.81 | Xanthoangelol_E |
| 47.08 | 0.00 | 38.81 | Isobavachalcone |
| 47.74 | 0.00 | 36.16 | Calceolarioside_B |
| 42.93 | 0.00 | 31.66 | Hesperetin |
| 41.04 | 0.00 | 30.82 | Neobavaisoflavone |
| 40.24 | 0.00 | 30.15 | Bavachinin |
| 37.74 | 0.00 | 39.44 | Beta-sitosterol |
| 37.35 | 0.00 | 27.45 | Luteolin |
