# Supplementary figures and images for: Senna makki and other active phytochemicals: Myths and realities behind covid19 therapeutic interventions
Source: PLoS One. 2022 Jun 14;17(6):e0268454. doi: 10.1371/journal.pone.0268454 (PMC9197063; doi:10.1371/journal.pone.0268454)

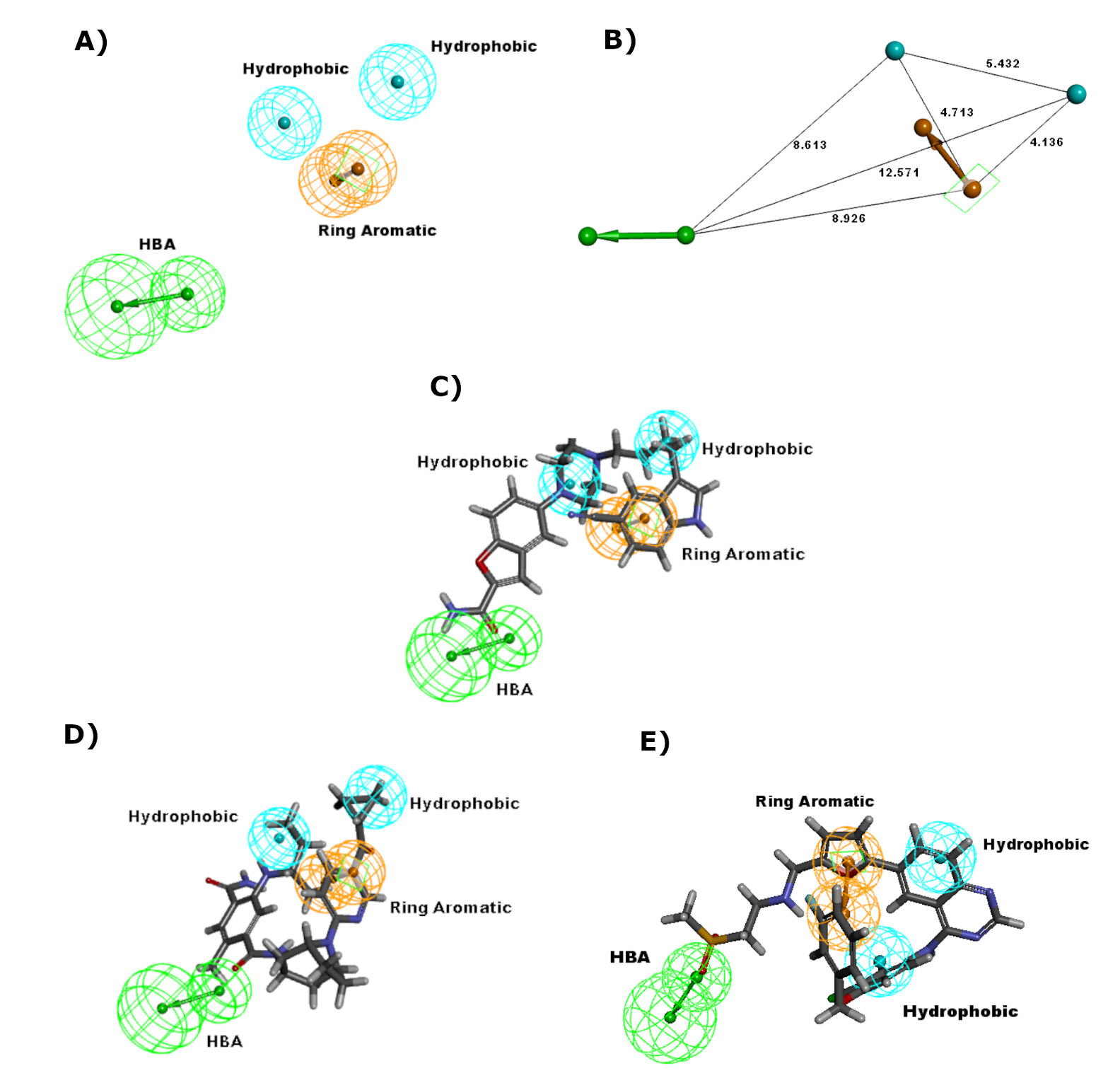

Supplement: S1 Fig — a) 3D-QSAR pharmacophore exhibits four common features consisting of 1 hydrogen bond acceptor (HBA), 2 hydrophobic (HYD), and 1 ring aromatic (RA) b) 3D-QSAR pharmacophore model with distance between chemical features. c) The most active medicinal compound; xanthoangelol_E from the training set mapped with the highest FitValue of 4.36 d) The top compound, vilazodone mapped against 3D-QSAR pharmacophore with the highest FitValue of 3.56 from the test set. e) The second top compound lapitinib with a FitValue of 3.54 mapped against 3D-QSAR pharmacophore from the test set. (TIF) [file pone.0268454.s007.tif]

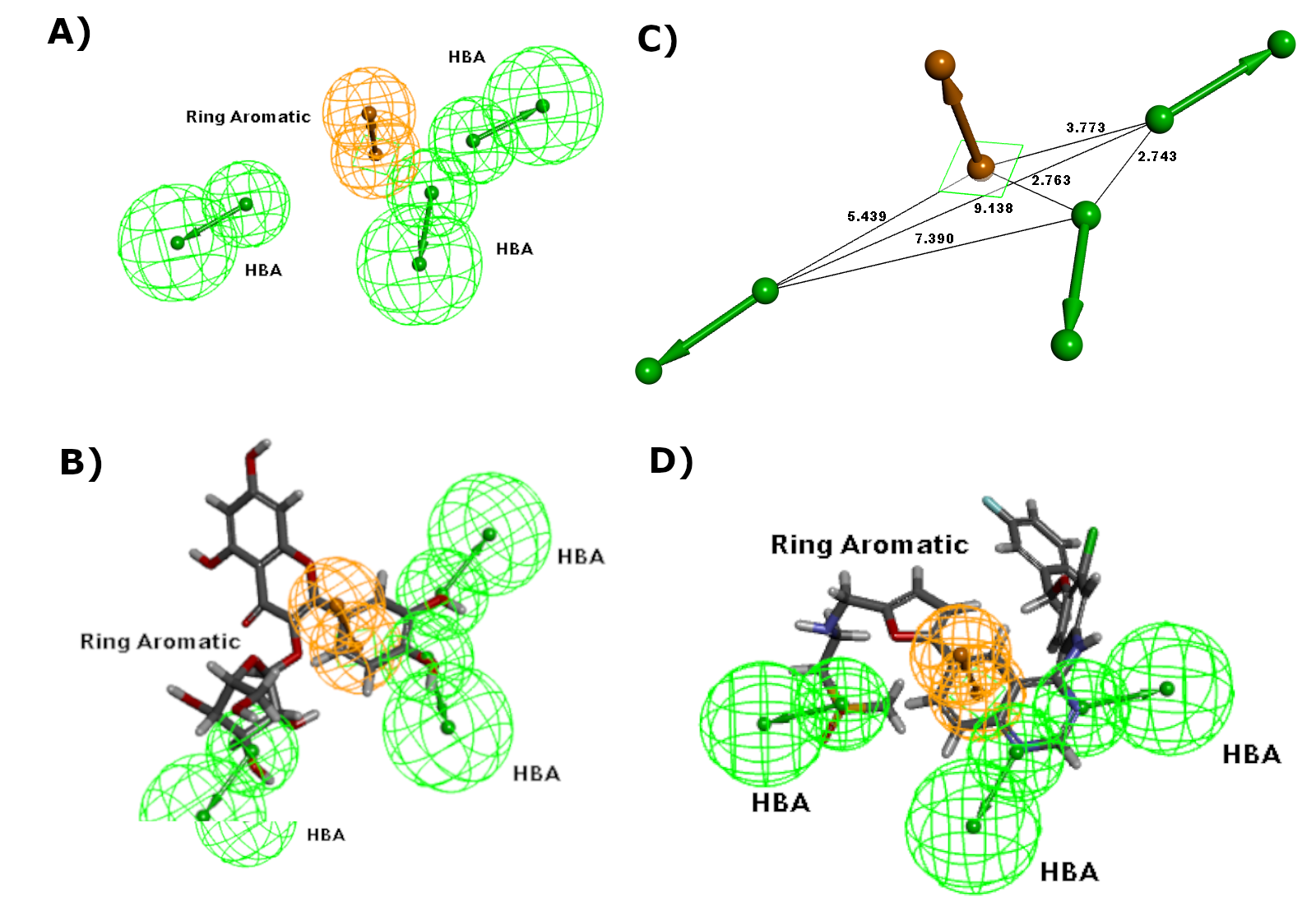

Supplement: S2 Fig — a) Common feature pharmacophore results of Senna compounds exhibit four common features consisting of 3 hydrogen bond acceptors (HBA) and 1 ring aromatic (RA) features b) Common feature pharmacophore model with distance between chemical features. c) Isoquercetin mapped the common feature pharmacophore with highest FitValue of 0.99 from the training set. d) Compound with lowest FitValue. (TIF) [file pone.0268454.s008.tif]

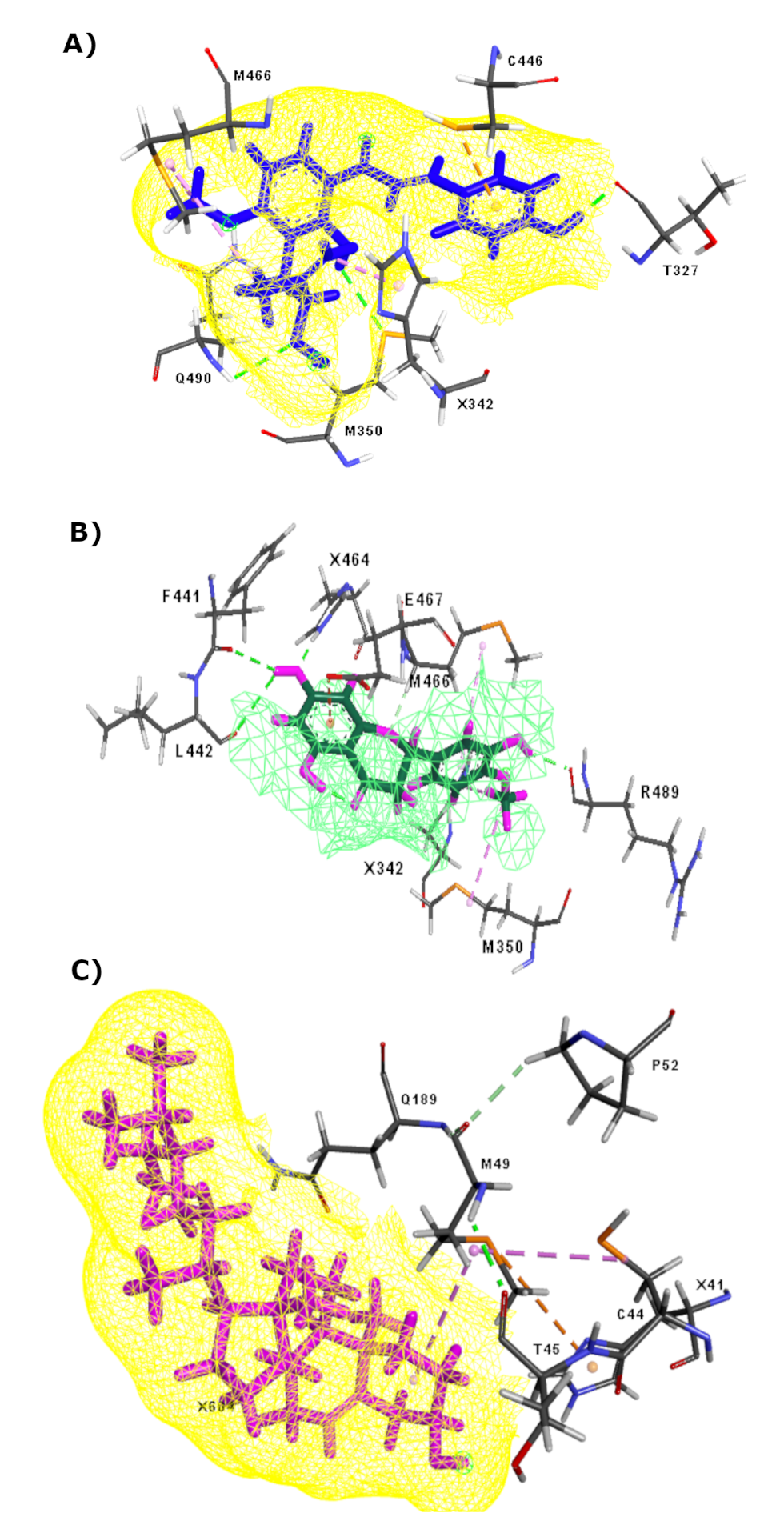

Supplement: S3 Fig — Preferred binding mode of active phytochemicals in the binding site of 3CLpro depicting two-dimensional (2D) docked complex with a) Xanthoangelol_E having an IC50 value 11.4 ±1.4 μM b) Hesperetin having an IC50 value 8.3 μM c) Beta-sitosterol with an IC50 value 1210 μM. (TIF) [file pone.0268454.s009.tif]

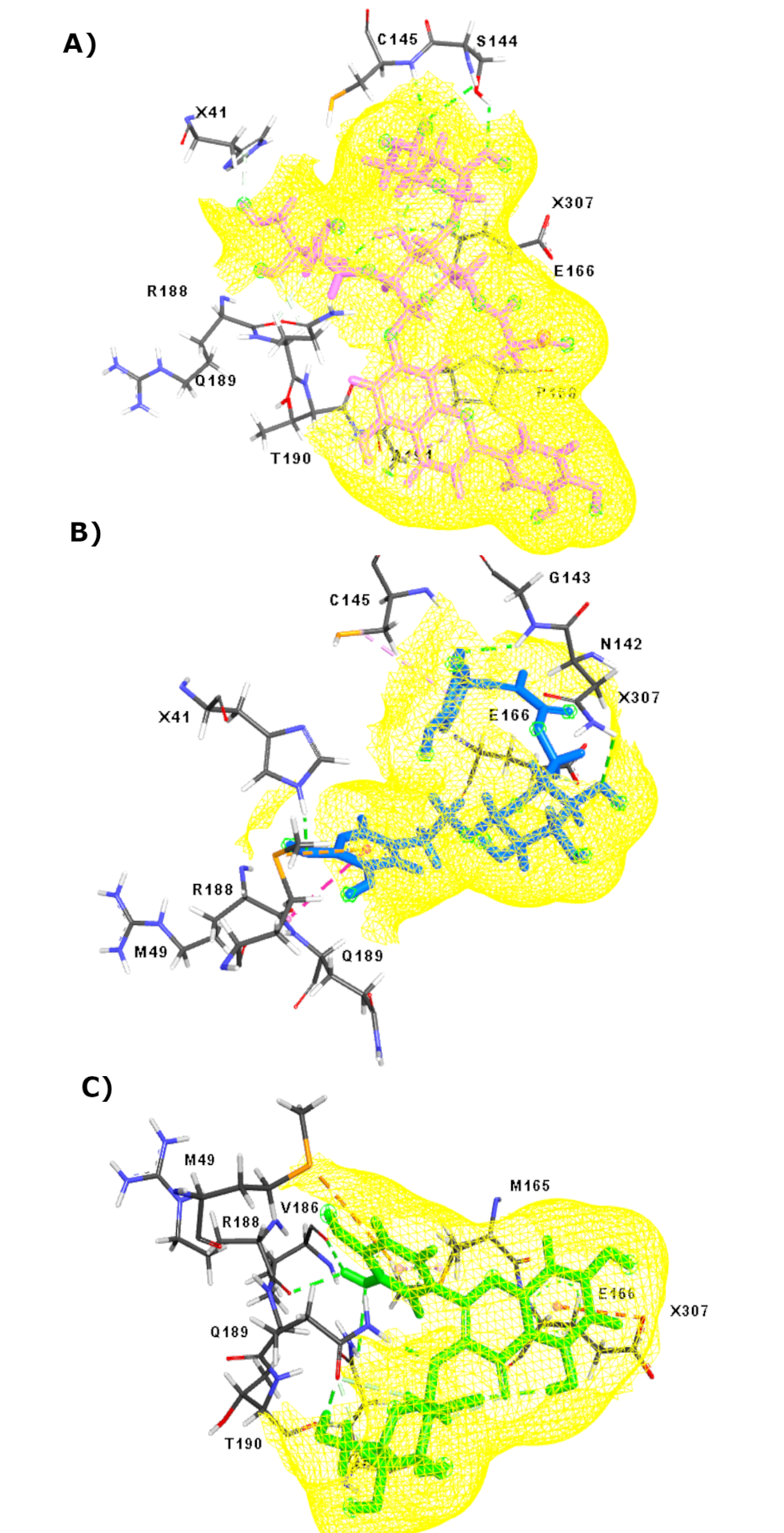

Supplement: S4 Fig — Preferred binding mode of active phytochemicals in the binding site of 3CLpro depicting two-dimensional (2D) docked complex with a) Luteolin-7-O-glucopyranoside b) Calceolarioside_B c) Isoquercetin. (TIF) [file pone.0268454.s010.tif]

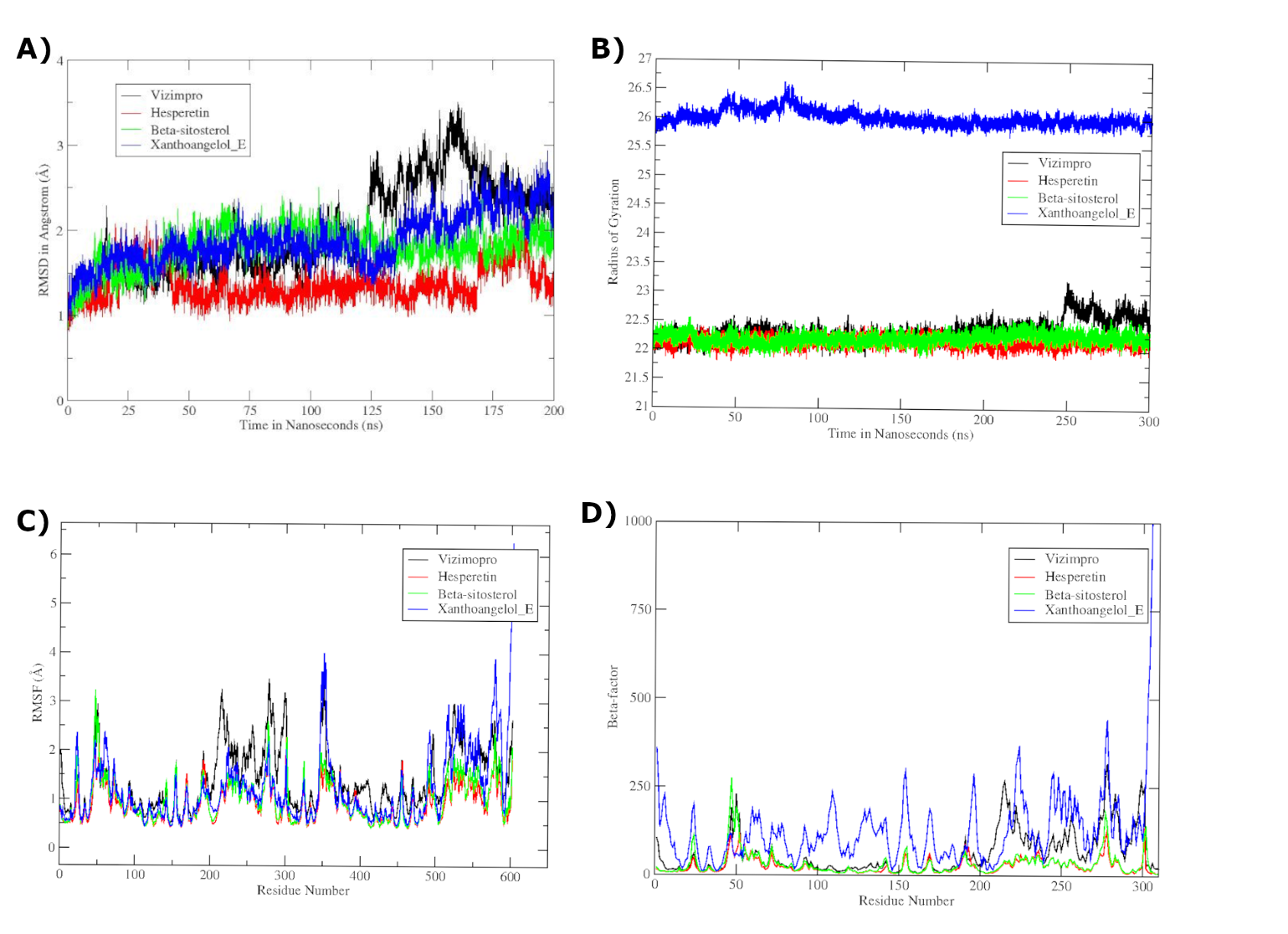

Supplement: S5 Fig — a) RMSD in complex with 3CLpro b) Radius of gyration c) RMSF of 3CLpro residues d) Beta-factor. (TIF) [file pone.0268454.s011.tif]

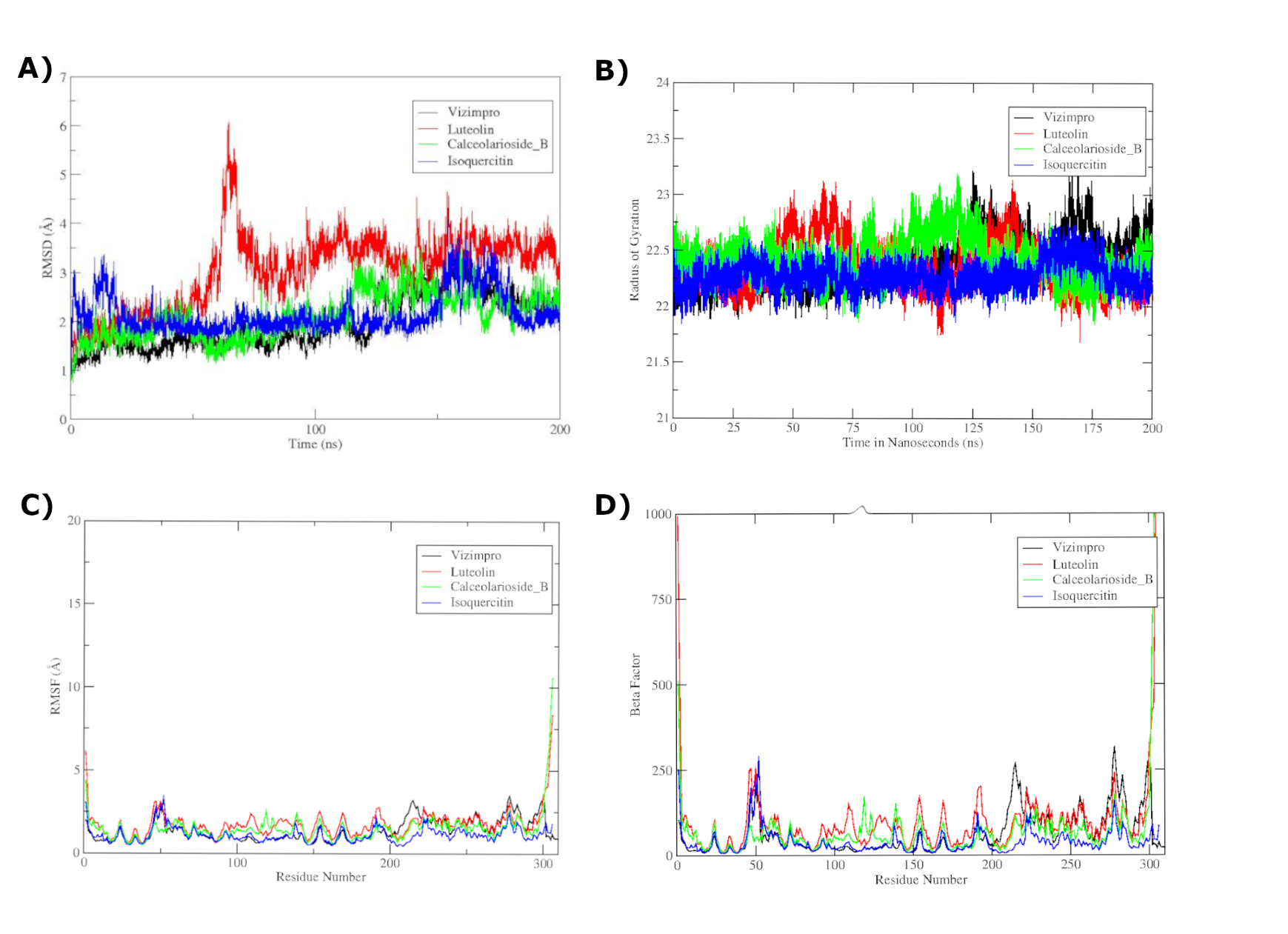

Supplement: S6 Fig — Insights into the MD simulations of vizimpro, luteolin, calceolarioside_B, and isoquercetin for the time period of 200 ns each complex a) RMSD in complex with 3CLpro b) Radius of gyration c) RMSF d) Beta-factor. (TIF) [file pone.0268454.s012.tif]

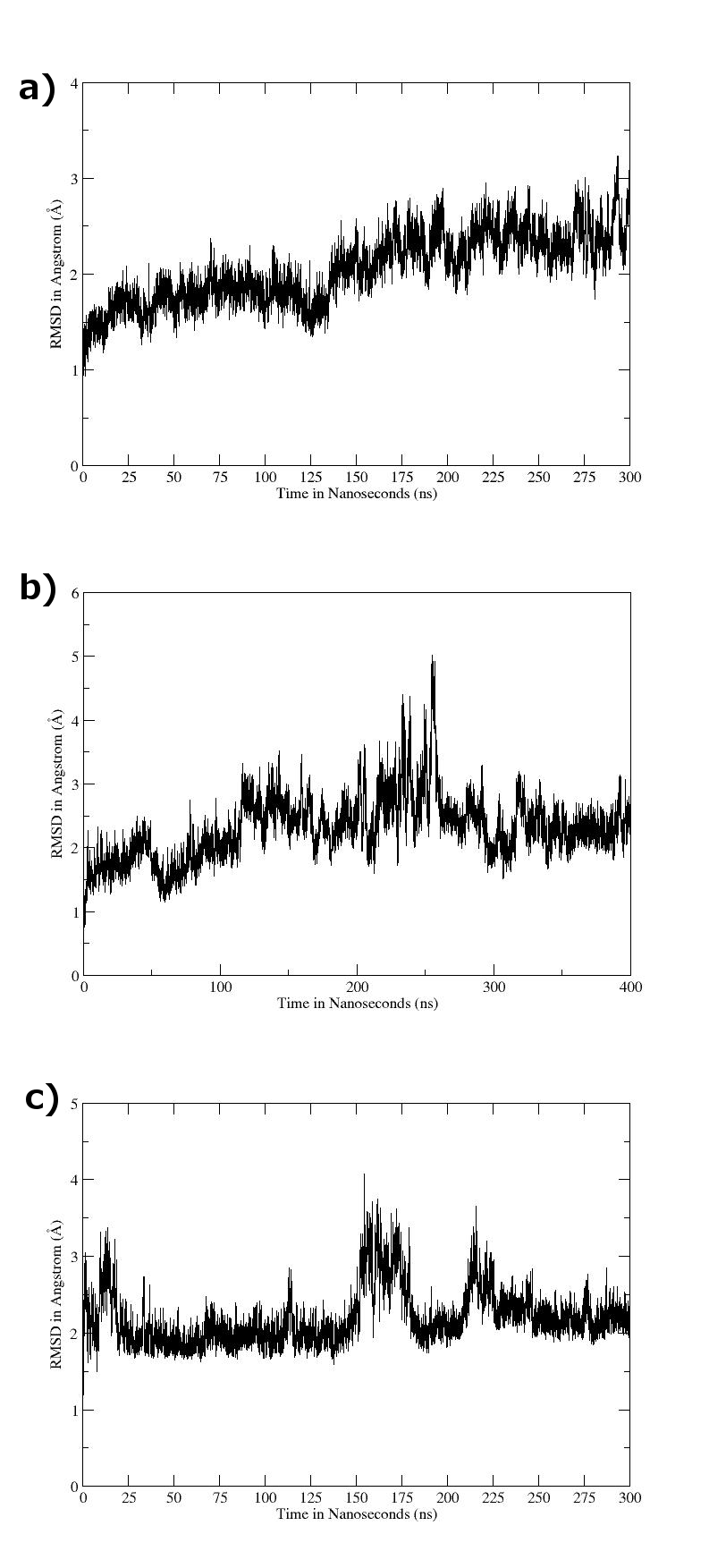

Supplement: S7 Fig — a) 100 ns of extended MD simulations of xanthoangelol_E b) 200 ns of extended MD simulations of calceolarioside_B c) 100 ns of extended MD simulations of isoquercetin. (TIF) [file pone.0268454.s013.tif]

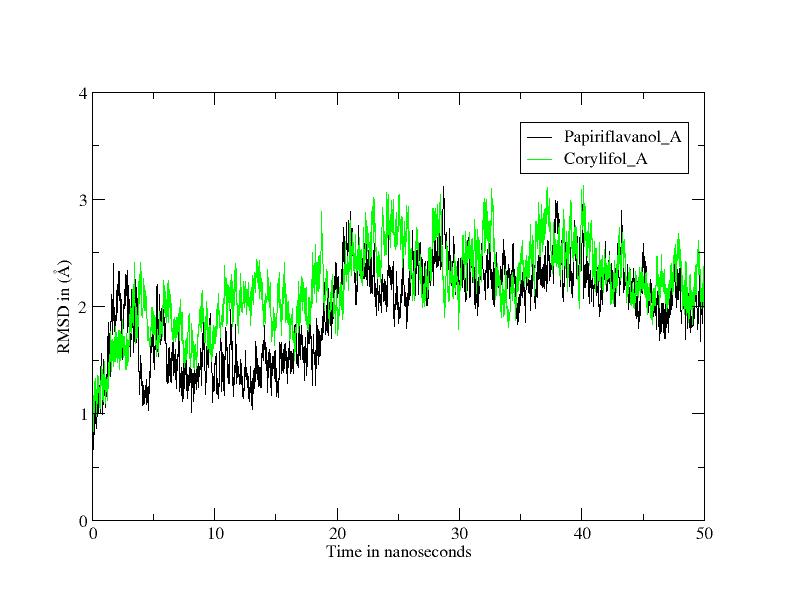

Supplement: S8 Fig — (TIF) [file pone.0268454.s014.tif]
